# Dependent autonomy: developing and refining a theoretical construct of managerial autonomy in healthcare

**DOI:** 10.1186/s12913-026-16096-4

**Published:** 2026-11-09

**Authors:** Mikael Ohrling

**Affiliations:** 1 Department of Learning, Informatics, Management and Ethics, Medical Management Centre, Karolinska Institutet, Stockholm, Sweden; 2 Stockholm Health Care Services, Region Stockholm, Sweden

**Keywords:** Decision space, Decentralisation, Organisational capacity, Accountability, Framework synthesis

## Abstract

**Introduction:**

Value-based healthcare (VBHC) and High-Value Health System (HVHS) frameworks identify components intended to support healthcare transformation. Their practical use may depend partly on organisational conditions affecting managerial authority. This study aimed to develop and refine dependent autonomy as a proposed theoretical construct concerning the practical exercise of delegated authority.

**Methods:**

A sequential two-stage design was used. Stage 1 involved secondary qualitative coding of published findings from four empirical programme studies, using directed content analysis informed by Decision Space Theory and organisational theory. An earlier scoping review served as a sensitising source. Stage 2 combined an external scoping review across three databases with hybrid deductive–inductive framework synthesis. Study selection and analytical interpretations underwent independent review in Stage 2. No formal critical appraisal was undertaken.

**Results:**

Stage 1 generated three themes: *enabled yet contingent autonomy*, *organisational capacity as a conditioning force*, and *coordination as integrative yet complexity-generating*. Stage 2 included 78 articles and yielded 482 analytical evidence statements. External findings informed refinements concerning deployable capacity, recognised managerial legitimacy and variation across functions, contexts and time. The synthesis proposed dependent autonomy as the organisationally conditioned actionability of formally delegated managerial authority. Four propositions concerned formal authority, organisational conditions, conditional consequences, and feedback and reconfiguration.

**Conclusions:**

Dependent autonomy may help examine how organisational arrangements enable or constrain managerial action, including in VBHC/HVHS implementation. The proposed framework distinguishes managers’ practical scope for action from improved organisational performance. Its explanatory value and proposed relationships require empirical examination; effects on patient value were not established.

**Supplementary Information:**

The online version contains supplementary material available at https://doi.org/10.1186/s12913-026-16096-4.

## Introduction

### Background

Healthcare transformation is often expressed through governance arrangements, digital infrastructure, care pathways, provider networks and payment models [1–3]. Value-based healthcare (VBHC) and High-Value Health System (HVHS) frameworks identify components intended to support coordination, improvement and patient value [2–4]. Their practical contribution depends on how they are used within healthcare organisations [5, 6].

Decision Space Theory distinguishes formally allocated authority, or de jure autonomy, from authority that managers can exercise in practice, or de facto autonomy [7, 8]. Information, resources, administrative support, coordination and accountability may enable managers to interpret situations and implement decisions. These arrangements may also constrain action when fragmented, burdensome or associated with unclear decision ownership [9–11]. Organisational dependencies may therefore make managerial discretion possible while also defining its limits.

Research on VBHC implementation identifies recurring difficulties involving information systems, resources, financial incentives and multidisciplinary collaboration [12]. Reviews of VBHC implementation and value-based purchasing similarly indicate that responses to financial incentives need to be understood in relation to programme design, provider characteristics and the wider context [13–15]. Research on innovation and quality improvement already recognises the importance of organisational context, leadership, local adaptation and interactions across levels [5, 6]. Building on this work, the present study addresses a specific conceptual problem: how organisational arrangements shape the practical exercise of delegated managerial authority. Further conceptual specification is needed to explain why similar formal mandates may afford different scopes for action and how the same organisational systems may enable or constrain that action.

This study proposes *dependent autonomy* as a theoretical construct describing the organisationally conditioned actionability of formally delegated managerial authority. It builds on Decision Space Theory [7, 8] by focusing on the organisational dependencies through which decision rights become practically exercisable. Its focus on managers acting close to service delivery complements broader accounts of embedded autonomy [16]. The preliminary construct emerged from a research programme examining managerial authority, organisational capacity and coordination in healthcare [17–21]. The programme provided contextual depth through its focus on a large public healthcare organisation and motivated further examination of the construct against external empirical evidence to explore its relevance beyond the original setting.

The article first presents the theoretical foundations and study design. It then reports the programme-derived themes and provisional construct, followed by the external review findings and the refined framework and propositions. The discussion considers the theoretical contribution, implications for healthcare governance, limitations and future research.

### Aim

The aim was to develop and refine dependent autonomy as a proposed theoretical construct for understanding how organisational systems enable or constrain the practical exercise of delegated managerial authority in healthcare organisations.

## Theoretical and analytical framework

### Decision space theory

Decision Space Theory conceptualises decentralisation through the choices available across functions such as finance, human resources, planning, service organisation and governance [7]. Its distinction between de jure and de facto autonomy makes clear that formal delegation and enacted authority are related but not identical. Later developments of Decision Space Theory emphasise that the practical use of delegated authority is shaped by the interaction between formal mandates, organisational capacity and accountability [8]. In the present study, this interaction provides the starting point for examining how formally allocated decision rights become actionable and how organisational conditions shape managerial autonomy, understood here as the scope managers have to make choices.

### Organisational systems as enabling and constraining arrangements

Organisational theory identifies structures, routines and technologies through which work is coordinated, and resources mobilised [9, 22]. Enabling bureaucracy supports users in handling their work, whereas coercive bureaucracy prioritises compliance and control [11]. This distinction informs examination of information and feedback systems, administrative and professional support, resource allocation, coordination, accountability and role structures. The analysis considers how their design and practical accessibility support or restrict managerial action.

### From the centralisation paradox to dependent autonomy

The centralisation paradox describes the coexistence and interdependence of decentralised authority and centrally coordinated enabling systems [23]. It highlights how local discretion may depend on shared information infrastructure, administrative support and system-wide coordination. A theoretical construct provides an abstract representation of a phenomenon for scientific analysis and requires a clear definition, scope and relationship to adjacent concepts [24]. In this study, dependent autonomy is proposed as a theoretical construct describing the organisationally conditioned actionability of formally delegated managerial authority. It elaborates Decision Space Theory’s distinction between formal authority and its practical exercise [7, 8].

### VBHC and HVHS as potential application context

VBHC and HVHS frameworks identify components intended to support high-value care, including outcome and cost measurement, digital infrastructure, integrated pathways and payment arrangements [2–4]. They provide a potential application context for the construct but did not determine eligibility for the external review.

## Methods

### Study design

**Fig. 1:**
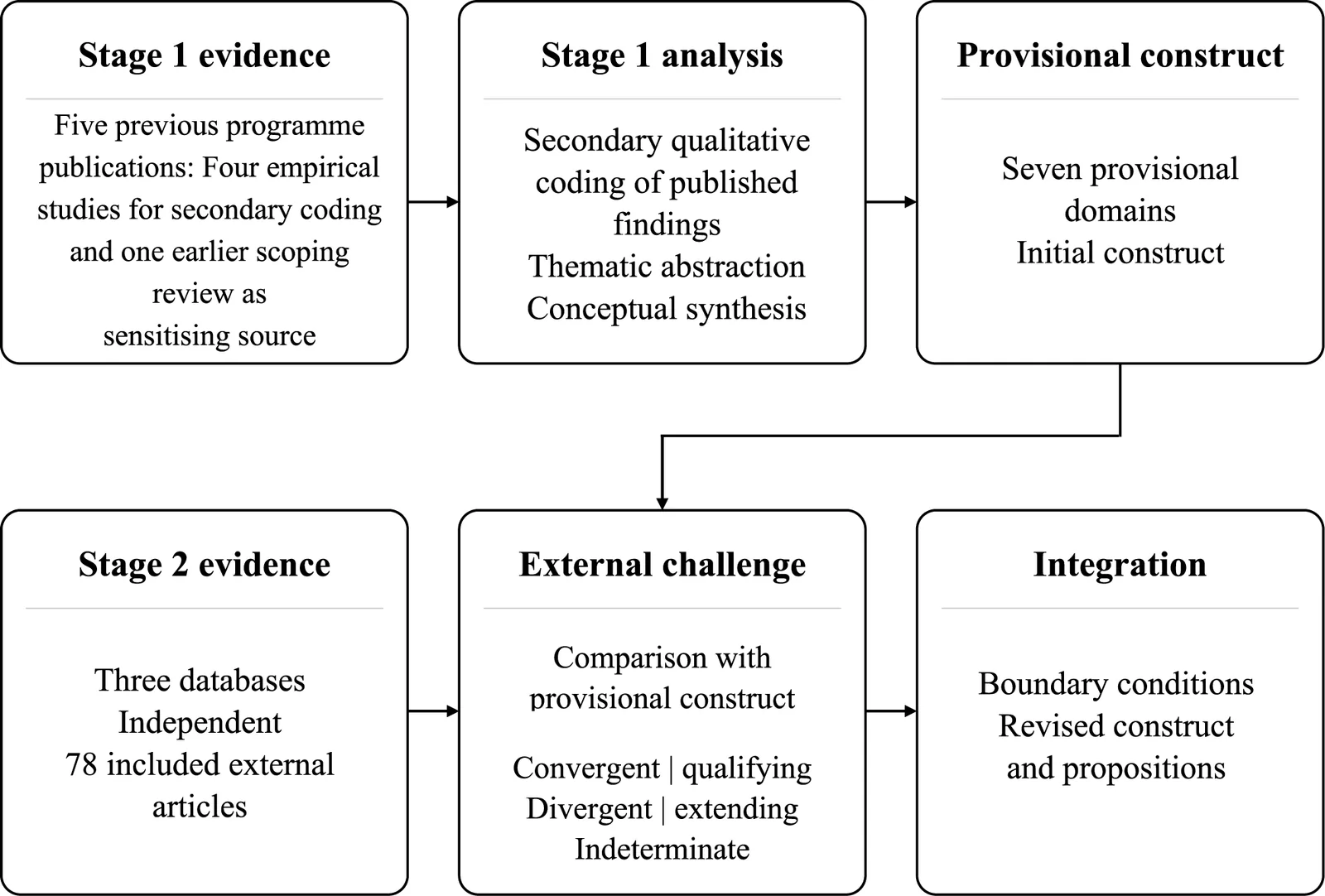
Sequential two-stage design for construct development and refinement. Programme-based construct generation in Stage 1 was followed by external examination and refinement in Stage 2. The earlier scoping review informed Stage 1 and was distinct from the external scoping review undertaken in Stage 2

The study used a sequential two-stage theory-building design (Fig. 1). Stage 1 combined secondary qualitative coding of published programme findings with conceptual synthesis to generate a provisional construct [25, 26]. Stage 2 identified external empirical evidence through a scoping review [27] and analysed it using hybrid deductive–inductive framework synthesis informed by best-fit framework synthesis [28]. This approach supported comparison with the provisional Stage 1 framework while allowing findings not adequately captured by it to inform revision and extension. The two corpora served distinct analytical purposes and were analysed separately.

### Stage 1 Programme-based construct generation

#### Material and selection rationale

Stage 1 drew on five publications from the same research programme, an earlier scoping review on decentralisation [21] and four empirical studies [17–20]. The publications were selected because they formed a coherent research programme examining managerial authority, organisational capacity and coordination in healthcare. The four empirical studies examined senior-management perspectives on decentralisation [17], clinical directors’ decision space [18], crisis management [19] and hybrid managerial arrangements [20] within the same healthcare organisation. Their selection reflected the complementary empirical insights they offered into the exercise of delegated authority. The shared organisational setting provided contextual depth, while variation across managerial levels and organisational circumstances supported comparison and construct generation. The empirical synthesis in this study used published findings from the results sections, tables, figures and supplementary material of the four empirical studies [17–20]. Primary interview and survey data were not reanalysed. Table 1 summarises the programme publications and their analytical roles.

**Table 1:** Programme publications and their analytical roles in Stage 1

Publication	Original data sources	Role in the present study
Ohrling et al. 2021a [21]	Scoping review; 24 included studies	Provided sensitising concepts concerning delegated authority, organisational capacity and accountability; not treated as an empirical case and not pooled with the four empirical studies.
Ohrling et al. 2021b [17]	Interviews with 9 senior managers	Informed the provisional construct by linking decentralised managerial authority to organisational capacity, central support and central–local responsibilities.
Ohrling et al. 2022a [18]	Interviews with 15 clinical directors	Informed the distinction between formal authority and practical scope for action through findings on the use and limits of delegated authority in clinical management.
Ohrling et al. 2022b [19]	23 interviews and weekly surveys sent to 108 clinical directors	Informed the analysis of how information infrastructure, resource mobilisation and coordination shape the exercise of managerial authority under crisis conditions.
Ohrling 2026 [20]	Two surveys of clinical directors (*n* = 80 and *n* = 92), with qualitative comments	Informed the interpretation of coordination as both enabling and constraining, linking hybrid managerial arrangements to uneven support, administrative demands and unclear responsibilities.

#### Programme analysis

The author (MO) conducted secondary qualitative coding of published programme findings using directed qualitative content analysis [29], informed by Decision Space Theory and organisational theory [7, 8, 11]. Coding was predominantly deductive at the category stage, while comparison, interpretation and construct development followed an iterative abductive process [30].

An analytical evidence statement was defined as a distinct published empirical observation concerning formal authority, managerial action or organisational conditions affecting the exercise of delegated authority. Eligible statements addressed decision rights, the use or non-use of authority, organisational enablers or constraints, coordination, accountability, decision ownership or proximal organisational consequences linked to authority. Method descriptions, background material and purely normative claims were not treated as empirical findings. Contextual, methodological and interpretive material retained in the coding inventory was identified by its analytical role. Duplicate observations were not treated as independent evidence, and outcomes without a relationship to authority were excluded from the empirical synthesis.

Statements were coded into seven theoretically informed categories: formal decision authority, enacted decision space, bounded autonomy, support systems, information infrastructure, central coordination and role ambiguity. The four analytical steps and their outputs are summarised in Table 2.

**Table 2:** Stage 1 analytical procedure from published findings to the provisional construct of dependent autonomy

Analytical step	Procedure	Analytical output
Identification and condensation	Relevant published findings were identified and condensed while preserving empirical meaning, organisational context and source links.	Analytical evidence statements linked to source publications.
Directed coding	Statements were coded into categories informed by Decision Space Theory and organisational theory.	Statements organised within seven analytical categories.
Cross-study comparison and thematic abstraction	Coded statements were compared within and across studies, considering variation across managerial levels, organisational circumstances and methods.	Broader themes describing how managerial autonomy was enabled, enacted and constrained.
Theoretical interpretation and conceptual synthesis	Themes were interpreted through iterative comparison with the theoretical framework, focusing on authority, capacity, accountability and enabling or constraining organisational systems.	A provisional conceptualisation of dependent autonomy for examination in Stage 2.

Ambiguous passages were coded according to their dominant meaning. Passages containing clearly separable meanings were divided into distinct analytical evidence statements and coded accordingly. Source links, coding decisions, and category definitions were documented in an audit trail. Supplementary File 1 provides category definitions and their theoretical derivation, together with worked examples tracing published findings through coding and thematic abstraction to conceptual interpretation.

The programme corpus was fixed, and saturation was not used to determine its size. The consolidated coding inventory preserved source links and recorded different types of analytical material. Its counts describe coding entries, not distinct eligible empirical findings, statistically independent observations, prevalence or conceptual importance. The descriptive inventory is presented in Supplementary File 1, while the Stage 1 analysis workbook records individual entries and their analytical roles. Findings contributed to the synthesis according to their substantive relevance, including their capacity to qualify recurring patterns.

Two senior researchers (CS and MB) critically reviewed the resulting Stage 1 analytical structure and provisional construct and provided constructive feedback. They did not independently code the programme material.

### Stage 2 external review and construct refinement

#### Review purpose and organisation

The preliminary construct developed in Stage 1 provided the starting point for Stage 2, termed the external review. A scoping review methodology was used to examine how organisational systems and conditions shape the translation of formally delegated managerial authority into action. This approach supported broad mapping across settings and study designs [31, 32], including findings that challenged, qualified or extended the preliminary construct. The review protocol and procedures are summarised in Supplementary File 2 A. Scoping-review methodology guided conduct, and reporting followed PRISMA-ScR and PRISMA-S [27, 33, 34].

The author (MO) and a second researcher (AB) independently conducted study selection. MO charted the data and conducted the initial analysis. AB verified charted information against full texts and independently reviewed coding, classifications and proposed refinements. A third researcher (UL) independently reviewed disputed cases and adjudicated unresolved differences in selection and analysis. AB and UL were independent of the empirical Stage 1 studies. Table 3 summarises the six review steps.

**Table 3:** Stage 2 review and analytical procedure

Analytical step	Procedure	Analytical output
Search and deduplication	Search three databases and remove duplicate records.	Records for screening.
Independent study selection	Apply eligibility criteria, reconcile decisions and link articles using shared data.	Included articles and documented decisions.
Charting and statement extraction	Extract study characteristics and findings with source locations.	Source-linked evidence matrix.
Deductive and inductive coding	Apply provisional domains and code findings outside the framework.	Domain assignments and additional concepts.
Comparison and classification	Compare contexts and classify relationships to the construct, including negative cases.	Patterns of convergence, qualification, divergence, extension and uncertainty.
Construct refinement and consensus	Link revisions to findings and resolve analytical disagreements.	Refined construct and documented revision decisions.

#### Eligibility criteria

Eligible articles were full-length, peer-reviewed primary empirical studies using qualitative, quantitative or mixed methods, assessable in English and addressing managerial or organisational decision rights in healthcare delivery. Findings had to relate authority to its practical exercise, organisational conditions or proximal organisational consequences. Studies limited to clinical or professional autonomy, and macro-level policy studies without provider- or manager-level findings, were excluded. The four Stage 1 empirical studies and publications using the same data were also excluded. Full criteria are provided in Supplementary File 2 A.

#### Search and deduplication

PubMed, CINAHL Complete via EBSCOhost and Web of Science Core Collection via Clarivate were searched on 31 August 2026 without publication-year restrictions. Four concept blocks were combined using AND: healthcare delivery, managerial actors or units, authority or decision space, and organisational conditions. Duplicates were removed using DOI, PMID and normalised-title matching. Full strategies, database-specific filters, search histories and result counts are provided in Supplementary File 2 A [34].

#### Study selection

The review register retained individual and reconciled decisions, with one primary reason for each full-text exclusion. Articles using shared or overlapping data were linked to avoid treating repeated findings as independent evidence. The selection flow and exclusion reasons are documented in Supplementary File 2 A.

#### Data charting and statement extraction

Charting covered study design, healthcare and governance context, managerial level, authority, organisational conditions and proximal consequences. Study characteristics are presented in Supplementary File 2B. Analytical evidence statements were distinct empirical findings extracted from results, tables, figures or supplementary material. Discussion interpretations were included only when explicitly linked to reported findings. Extraction preserved meaning, context and source locations. Formal critical appraisal and risk-of-bias assessment were not undertaken because the review mapped concepts and relationships for construct development [27, 33]. The emphasis was on identifying recurring patterns, contextual variation and findings that could qualify or extend the provisional framework across heterogeneous study designs, rather than on estimating effects or establishing the certainty of the proposed relationships [32].

#### Deductive and inductive coding

Hybrid deductive–inductive framework synthesis enabled comparison with the provisional construct while retaining findings outside it [28]. The seven Stage 1 analytical categories provided the initial organising domains. Some labels were broadened, informed by the programme findings and their theoretical interpretation, to encompass both enabling and constraining arrangements across healthcare contexts. For example, “role ambiguity” became “accountability, decision ownership and role clarity”, reflecting the underlying question of how responsibilities and decision rights are allocated and aligned. The mapping and rationale are presented in Supplementary File 1, Table S1.4. Statements were assigned a primary domain and, where appropriate, secondary domains. Findings without a clear fit were retained and coded inductively to inform possible additions or revisions to the framework. Domain definitions and worked examples are provided in Supplementary File 2C.

#### Comparison and classification

Extracted empirical findings from the external review were compared across articles, linked reports, healthcare settings, managerial levels and study designs to examine recurring relationships, contextual variation, negative cases and rival explanations [28]. The components and proposed relationships of the provisional construct developed in Stage 1 provided the reference for classification. Findings were classified as convergent when they supported these components or relationships, qualifying when they specified conditions or limits, divergent when they contradicted a proposed relationship or supported a rival explanation, and extending when they introduced additional conditions, mechanisms or relationships. Findings whose relationship to the construct could not be determined confidently were classified as indeterminate. These study-specific classifications were recorded at statement level and summarised at article level, allowing each article to contribute findings to several classes. Definitions, descriptive distributions and worked examples are provided in Supplementary File 2C.

#### Construct refinement and consensus

External findings were used to reconsider the components of the provisional construct developed in Stage 1 and determine whether they should be retained, qualified, extended or reorganised. Each revision decision was linked to the articles and evidence statements that informed it, checked against the original sources and finalised through the review, consensus and adjudication procedures described above. Supplementary File 2 C summarises the revisions and provides illustrative links to supporting evidence. The full revision matrix is retained in the Stage 2 analysis workbook.

### Ethical considerations

The present study forms part of a broader research programme on healthcare management whose research plan was reviewed and endorsed by the Stockholm Regional Research Ethics Board (DNr 2018/98 − 31/5) [17, 18, 20, 21]. The crisis-management study [19] was conducted within a separately reviewed project approved by the Swedish Ethical Review Authority (Dnr 2020–01521). The present synthesis analysed published findings and involved no participant recruitment or reanalysis of individual-level primary data. Consent to participate was therefore not applicable. Further details of ethical approvals and consent procedures for the original studies are provided in the respective publications and in the Declarations section of this article.

## Results and conceptual development

### Stage 1 Programme-derived themes and provisional construct

The Stage 1 synthesis generated three overarching themes across the seven analytical categories: *enabled yet contingent autonomy*, *organisational capacity as a conditioning force*, and *coordination as integrative yet complexity-generating*. Together, these themes informed the provisional conceptualisation of dependent autonomy.

Source-linked examples of coding, cross-study comparison and thematic abstraction are presented in Supplementary File 1. The original coding inventory is reported separately in that supplement.

#### Enabled yet contingent autonomy

Formal managerial responsibilities encompassed budgets, staffing, local operations, care quality, work environment and organisational performance [17–20]. Managers exercised authority through local adaptation, service development and changes to staffing and work processes, including during crisis conditions. Central policies, financial restrictions, purchaser contracts and decisions at other organisational levels bounded their practical discretion [18, 19]. In the hybrid management study, formal authority was retained while additional coordinating roles introduced uncertainty about responsibilities and decision boundaries [20].

#### Organisational capacity as a conditioning force

HR and financial expertise, leadership development, legal advice, improvement programmes, digital infrastructure and operational support helped managers exercise delegated responsibilities [17–20]. Their contribution depended on availability and practical usefulness. In the hybrid management study, support improved in some areas but remained uneven and dependent on individual contacts [20]. Information systems supported overview, coordination and adaptation, while insufficient feedback, fragmented information and weak digital support constrained planning [18–20].

#### Coordination as integrative yet complexity-generating

Coordination supported common priorities, organisational overview and integration across interdependent services. During crisis management, this included staffing, logistics, information sharing and collaboration across organisational boundaries [17–20]. The hybrid management study also documented additional interfaces, overlapping responsibilities, blurred boundaries between managerial levels and administrative demands [20]. Coordination was described as supporting local action but also adding complexity when mandates, accountability and decision ownership were unclear.

#### Provisional conceptualisation of dependent autonomy

Across the three themes, the reported findings described formally delegated responsibilities alongside variation in managers’ practical scope for action. Access to usable resources and information, administrative and professional support, and coordination arrangements were described as relevant to the exercise of authority [17–20]. These findings informed a proposed provisional conceptualisation of dependent autonomy, describing how organisational conditions may shape the practical exercise of formally delegated managerial authority.

### Stage 2 External review and construct refinement

#### Search and deduplication

The searches identified 826 records: 494 from PubMed, 90 from CINAHL Complete and 242 from Web of Science Core Collection. Removal of 216 duplicates left 610 unique records for screening (Supplementary File 2A).

#### Study selection

Following screening and full-text assessment, 78 articles were included [8, 35–111]. Some articles reported findings from shared or overlapping data. The selection flow and full-text exclusion reasons are presented in Supplementary File 2A, and all included articles are described in Supplementary File 2B.

#### Data charting and statement extraction

The included articles were published between 1994 and 2026 and predominantly concerned settings in Africa (*n* = 43) and Asia (*n* = 16). Most used qualitative, documentary, case-based or participatory designs (*n* = 55), followed by mixed-methods (*n* = 14) and quantitative designs (*n* = 9). The most common analytical levels were regional or subnational systems (*n* = 29), organisations, hospitals or networks (*n* = 18), and multilevel governance systems (*n* = 17). Detailed article characteristics are presented in Supplementary File 2B. Charting yielded 482 analytical evidence statements linked to their sources. These are summarised in Supplementary File 2C and documented in full in the Stage 2 analysis workbook.

#### Deductive and inductive coding

Findings addressed all seven initial domains: formal decision authority, enacted decision space, bounds and contingencies, support and resource systems, information and feedback infrastructure, coordination, and accountability, decision ownership and role clarity (Supplementary File 2C, Table S2C.1). Coding further specified recognised managerial legitimacy, informal influence and relational infrastructure [72, 84, 98, 105]. Capacity-related findings distinguished the presence of resources from their practical availability for managerial use [43, 86].

#### Comparison and classification

The matrix contained 226 convergent, 88 qualifying, 10 divergent, 155 extending and three indeterminate statements. These counts describe the coded material, rather than evidential strength or prevalence. Definitions and descriptive distributions are presented in Supplementary File 2C, Table S2C.2; the complete statement-level classifications are retained in the analysis workbook.

Convergent findings supported the distinction between formal authority and enacted decision space [8, 70, 87] and linked managerial action to usable resources, information, support and coordination [43, 84, 91]. Qualifying findings specified variation by function and managerial level [64, 70, 91], while historical evidence documented expansion and contraction of decision space over time [73]. Central coordination and accountability could enable local action or constrain it through delays, restrictive rules and administrative demands [70, 91]. Extending findings identified legitimacy, trust and professional and political relationships as conditions shaping the use of formal authority [72, 77, 84, 105].

Findings also identified limits to the relationship between managerial autonomy and organisational performance. In an Indian immunisation study, greater measured decision space was associated with less improvement in the coverage gap [54]. A Zambian study found no statistically significant difference in autonomy between performance-based financing and enhanced input financing [48], leaving the additional contribution of performance incentives uncertain. Other findings described workarounds that sustained local activity but could shift resources towards revenue-generating services or involve departures from formal procedures [41, 91].

#### Refinements to the provisional construct

External findings addressed all seven provisional domains while specifying their scope and content. Resource-related findings distinguished the presence of resources and support from managers’ ability to access and use them. Coordination-related findings identified the contribution of working relationships alongside formal arrangements. Findings concerning professional credibility, acceptance of coordination mandates and competing stakeholder expectations linked legitimacy to support, coordination and contextual constraints.

The revisions retained the seven-domain structure and made recognised managerial legitimacy explicit as a cross-cutting condition (Table 4). Formal authority was specified as function-specific and distributed, while enacted decision space encompassed use, non-use and adaptation. Other refinements specified the practical availability of resources and support, usable information and feedback, relational coordination, and accountability aligned with decision rights. Contextual and temporal variation was incorporated into the domains. Supplementary File 2C, Table S2C.3, links these revisions to illustrative findings.

**Table 4:** Refinements to the provisional framework following Stage 2

Component	Result of refinement
Formal decision authority	Specified as function-specific and distributed across organisational levels and actors [64,70,98].
Enacted decision space	Broadened to encompass adaptation, non-use and workarounds [8, 41, 101].
Bounds and contingencies	Extended to include temporal, political and professional conditions [73, 77, 105].
Support and resource systems	Reformulated as deployable organisational capacity, distinguishing available resources from resources managers could use [43, 86].
Information and feedback infrastructure	Extended to include feedback and learning [89, 91].
Coordination	Broadened to include relational infrastructure [72, 98].
Accountability, decision ownership and role clarity	Specified through role clarity, decision ownership and escalation arrangements [82, 91, 98].
Recognised managerial legitimacy	Made explicit as a condition operating across the seven domains [84, 101, 105].

### Conceptual synthesis

The Stage 1 synthesis proposed dependent autonomy to describe how organisational conditions shaped managers’ practical scope for exercising formally delegated authority. The external synthesis refined this formulation by distinguishing the presence of resources and support from their practical usability, extending coordination to include working relationships, and making recognised managerial legitimacy explicit as a cross-cutting condition. It also specified variation across functions, managerial levels, contexts and time.

The integrated synthesis retained dependent autonomy as a proposed theoretical construct describing the organisationally conditioned actionability of formally delegated managerial authority. The refined formulation emphasised the combined role of decision rights, deployable resources and expertise, usable information, coordination, aligned accountability and recognised legitimacy. These conditions were represented as potentially enabling or constraining the exercise of authority. The formulation distinguished the practical capacity to act from whether action advanced organisational or public-value objectives.

The conceptual synthesis generated four propositions for empirical examination:

#### Proposition 1

*Formal authority and practical actionability.*

Formally delegated managerial authority is function-specific and distributed across organisational levels and actors. Its formal allocation does not determine whether or how managers can exercise it.

#### Proposition 2

*Organisational conditions of actionability.*

The practical exercise of delegated authority depends on managers’ ability to mobilise resources and support, use relevant information, coordinate interdependent activities and act with recognised legitimacy within accountability arrangements aligned with their responsibilities and decision rights.

#### Proposition 3

*Conditional organisational consequences.*

Actionable authority may facilitate managerial action across interdependent services. Its contribution to coordination and patient-flow processes depends on how authority is exercised and on organisational and contextual conditions. Greater scope for action does not necessarily produce better organisational performance.

#### Proposition 4

*Feedback and reconfiguration.*

Feedback from managerial action, non-action and informal adaptations may lead to changes in decision rights and organisational arrangements. These changes may expand, restrict or redistribute the practical scope of managerial authority over time.

**Fig. 2:**
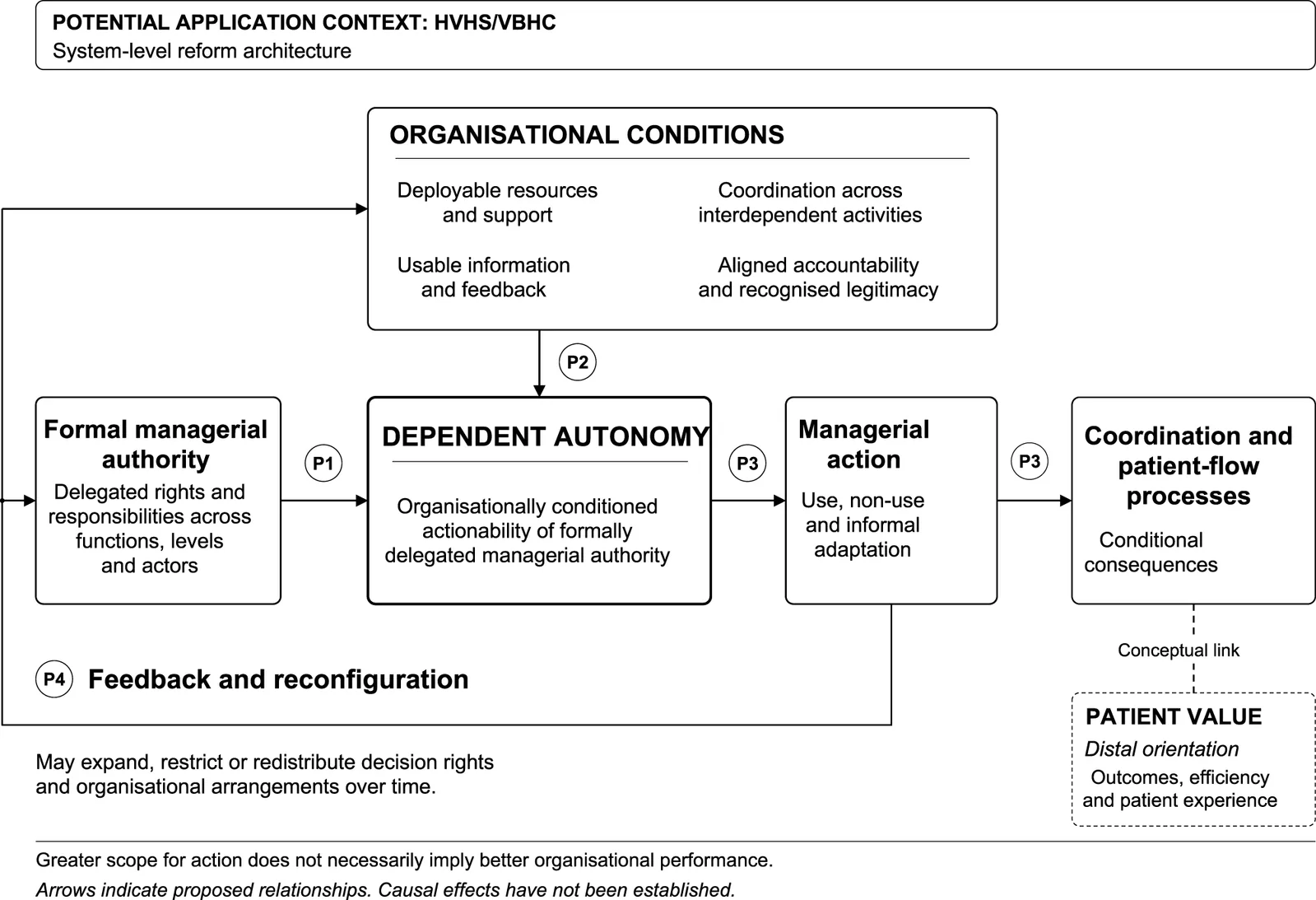
Proposed integrated conceptual framework of dependent autonomy. **P1**–**P4** refer to the propositions in the text. Solid arrows indicate proposed relationships, while the dashed line represents a distal conceptual link

Figure 2 presents the proposed integrated framework. The propositions concern managerial and organisational processes. Their causal relationships and effects on patient value were not tested.

## Discussion

The sequential synthesis informed the development and refinement of dependent autonomy. Stage 1 provided a programme-derived conceptualization, and Stage 2 broadened its empirical basis and specified the practical usability of capacity, the contribution of legitimacy and relationships, and variation across functions and contexts. The framework distinguishes managers’ practical scope for action, their exercise of authority and the consequences of that exercise.

### Theoretical contribution of dependent autonomy

Dependent autonomy is proposed as a theoretical construct describing the organisationally conditioned actionability of formally delegated managerial authority. It offers an elaboration of Decision Space Theory’s account of authority, capacity and accountability [7, 8], focusing on the arrangements through which decision rights become practically exercisable. Interpretation of mandates, resource mobilisation, use of information and securing cooperation are possible processes underlying this actionability. The synthesis identifies them for further investigation but does not establish their causal operation.

This formulation preserves managerial agency. Managers may interpret similar mandates differently, refrain from using available authority, negotiate support or act beyond formal boundaries. Findings concerning non-use of disciplinary authority and differences between formal and informal influence illustrate why neither formal mandates nor observed action alone adequately describe managers’ scope for action [101, 105]. Organisational conditions may shape available choices without determining how managers act.

### Organisational capacity, coordination and legitimacy

The programme findings suggested that organisational systems could simultaneously enable and constrain managerial action [17–20], consistent with the distinction between enabling and coercive bureaucracy [11]. Information, administrative support, leadership development and coordination helped managers exercise authority, while administrative burden, weak feedback and unclear decision ownership restricted its use. Stage 2 further distinguished resources or infrastructure that were nominally available from capacity that managers could mobilise. Delayed funding, spending restrictions and weak connections between information and decision routines illustrated this distinction [43, 86, 89]. Table 5 provides examples of relevant organisational arrangements.

**Table 5:** Examples of organisational systems relevant to dependent autonomy

System type	Examples
Information systems	Electronic health records, dashboards, analytics, outcome and capacity data
Administrative systems	Human resources, finance, procurement, legal and operational support
Coordination systems	Management meetings, operational care-pathway routines, escalation arrangements and cross-unit forums
Development systems	Leadership training, improvement programmes and professional development
Governance systems	Performance management, accountability structures and reporting routines

Note. These overlapping system types illustrate organisational arrangements. They are not additional analytical domains

This duality connects with research on audit and performance management, which describes tensions between accountability, measurement and operational priorities, including attention to measurable targets and demonstrable compliance [112–114]. Research on health information technology similarly emphasises interactions between technical design, organisational processes and working practices [115, 116]. These perspectives support examining how systems are used and experienced, since their presence alone does not establish their contribution to managerial action.

Coordination may expand practical options through shared information, expertise and escalation, or narrow them through restrictive approvals and overlapping responsibilities [70, 91, 98]. Coordination arrangements are conditions of action; coordination achieved between services is a possible consequence. Legitimacy concerns whether managerial decisions are accepted and cooperation secured, beyond possession of formal rights and resources [84, 101, 105]. Its explicit inclusion across domains develops an issue already present in the programme interpretation.

The external findings also caution against equating greater actionability with better performance [41, 54]. The consequences of exercising authority remain a separate empirical question. Evidence of changing decision space supports attention to temporal conditions [73], but the feedback and reconfiguration processes proposed in P4 require further investigation.

### Relationship to existing concepts and scope

The expression dependent autonomy has been used in other organisational contexts, including outsourcing [117]. The contribution proposed here concerns its specification for delegated managerial authority in healthcare. The centralisation paradox characterises a governance-level tension [23], whereas dependent autonomy is proposed to focus on the interface between organisational systems and managers’ practical scope for action. The concepts therefore address related questions at different analytical levels. Table 6 shows how this focus relates to established concepts without implying that it provides a wholly separate explanation.

**Table 6:** Conceptual positioning of dependent autonomy

Concept	Primary focus	Complementary focus of dependent autonomy
De jure autonomy [7, 8]	Formally allocated decision rights.	Organisational conditions under which those rights become practically exercisable.
De facto autonomy [7, 8]	Practical scope and exercise of authority.	Organisational dependencies that may help explain variation in actionability.
Organisational capacity [8, 9, 22]	Resources, competence, infrastructure and support.	Whether capacity can be mobilised to exercise decision rights and responsibilities.
Relational autonomy [118]	Autonomy constituted and exercised through social relationships.	Relationships involved in exercising delegated managerial authority.
Bounded autonomy [119]	Local adaptation within wider strategies and governance constraints.	Enabling and constraining dependencies affecting usable managerial discretion.
Embedded autonomy [16, 120]	Autonomy in relation to institutional and relational embeddedness.	The proximate interface between organisational systems and managers exercising decision rights.
Centralisation paradox [23]	Interdependence between decentralised authority and central coordination.	How coordination and other dependencies shape the practical exercise of managerial authority.
Dependent autonomy*	Organisationally conditioned actionability of formally delegated managerial authority.	Brings decision rights, deployable capacity, information, coordination, accountability and legitimacy into a common analytical focus.

Note. The comparison identifies complementary emphases. It does not imply that related theories exclude organisational conditions or managerial agency. *Dependent autonomy denotes the construct proposed in this study

Its proposed scope includes managers with identifiable decision rights within decentralised, centralised or hybrid arrangements. Informal influence is relevant when it enables, overrides or reshapes those rights. The construct does not encompass all professional discretion or organisational agency, and its applicability beyond the settings represented in the synthesis requires further examination.

### Implications for healthcare transformation

VBHC and HVHS provide a potential application context through their emphasis on outcome and cost information, integrated care and system-level infrastructure [2–4]. A dashboard or care pathway may change managerial information or expectations without supplying the resources, decision rights or cooperation needed to respond. Dependent autonomy directs attention to these connections between reform architecture and operational action. The external review was not restricted to VBHC/HVHS and does not demonstrate their implementation effects. Coordination and patient-flow processes are proximal areas for investigation. Patient value remains a distal conceptual orientation. Effects on patient outcomes, efficiency and experience were not tested and require separate empirical assessment.

### Implications for research and practice

Research should distinguish formal decision rights, organisational conditions, practical scope for action, enacted decisions and their consequences. Defining dependent autonomy solely through support systems or successful action would obscure these relationships. Comparative studies could examine similar mandates under different organisational arrangements. Longitudinal studies of decision episodes could investigate how resources, information, cooperation and feedback alter actionability, including cases of non-use, override and informal adaptation.

For practice, the framework offers questions about which decisions are authorised, what resources and support can be mobilised, whose cooperation is required, and where responsibility for implementation and escalation lies. These questions may help identify mismatches between responsibility and practical scope for action. Their usefulness for organisational diagnosis and improvement remains to be evaluated; the framework does not prescribe uniformly greater discretion or additional organisational systems.

### Strengths, reflexivity and limitations

The two-stage design combined contextual depth from the programme studies with a broader external empirical base. Published interview and survey findings from different managerial levels provided complementary perspectives on delegated authority and its organisational conditions within the programme [17–20]. The external review examined findings that supported, qualified, challenged or extended the provisional construct. The two corpora were analysed separately. In Stage 2, independent study selection, source verification and analytical review provided additional scrutiny, with unresolved disagreements referred for adjudication. Both external researchers were independent of the empirical Stage 1 studies.

Stage 1 nevertheless drew on four empirical studies from one research programme within a Swedish healthcare organisation. The author’s involvement provided contextual knowledge but also created a risk of confirmation bias. Two senior researchers critically reviewed the provisional analytical construct but did not independently code the material. The external review broadened the empirical basis and introduced independent scrutiny; it did not constitute an independent replication or validation of the programme synthesis.

The external search covered three databases and English-language, peer-reviewed publications. Requiring explicit authority-related and organisational-condition concepts may have missed relevant work using other terminology, while unavailable full texts may have introduced selection bias. The concentration of articles in African and Asian settings limits the basis for assessing transferability to other health systems. Heterogeneous, predominantly qualitative evidence supports contextual interpretation but does not establish the prevalence of relationships.

Both stages depended on published findings, interpretations and reporting detail. Individual-level primary data were not reanalysed, and emerging interpretations could not be explored through additional participant data. Formal critical appraisal and risk-of-bias assessment were not undertaken in Stage 2, limiting assessment of the methodological credibility of individual findings and confidence in the proposed relationships. Source verification and analytical consensus do not remove these limitations.

The explanatory value of dependent autonomy relative to decision space, organisational capacity and embedded autonomy remains to be assessed. The proposed relationships require empirical examination across healthcare systems, managerial levels and organisational configurations. Effects on coordination, patient-flow processes and patient value have not been established.

## Conclusions

This study proposes dependent autonomy as a theoretical construct describing the organisationally conditioned actionability of formally delegated managerial authority. Programme-based synthesis and an external scoping review highlighted deployable capacity, usable information, coordination, recognised legitimacy and aligned accountability as conditions relevant to the practical exercise of authority.

The construct draws attention to an autonomy–coordination tension: managers need discretion to adapt services to local needs, while exercising it through systems that coordinate, standardise and regulate action. These arrangements may enable discretion while defining its limits. Managers retain agency, and greater scope for action does not necessarily improve organisational performance.

With VBHC/HVHS as a potential application context, the framework offers a basis for investigating how organisational arrangements connect reform ambitions with managerial action, coordination and patient-flow processes. The proposed relationships require empirical testing. Patient value remains a distal conceptual orientation; effects on patient outcomes, efficiency and experience were not established.

## Supplementary Information

Below is the link to the electronic supplementary material.

Supplementary Material 1: Supplementary File 1. Stage 1 analytical procedure and traceability. Category development, descriptive coding inventory, worked examples, thematic abstraction and mapping to the provisional Stage 2 domains.

Supplementary Material 2: Supplementary File 2 A. Stage 2 review scope, searches and study selection. Protocol summary, detailed eligibility criteria, database search strategies and histories, search yields, selection flow and full-text exclusion reasons.

Supplementary Material 3: Supplementary File 2B. Descriptive characteristics of the 78 included articles. Bibliographic details, settings, designs, analytical levels and substantive focus.

Supplementary Material 4: Supplementary File 2 C. Stage 2 analysis and construct refinement. Coding domains, relationship classifications, descriptive distributions, worked examples, documented refinements and propositions.

## Data Availability

Stage 1 used published findings from four empirical programme studies [17–20], with an earlier scoping review serving as a sensitising source [21]. Supplementary File 1 documents category development worked analytical examples, the descriptive coding inventory and the transition to the provisional Stage 2 domains.For Stage 2, Supplementary File 2A documents the review scope, eligibility criteria, search strategies, selection flow and exclusion reasons. Supplementary File 2B provides the descriptive characteristics of all 78 included articles. Supplementary File 2C presents the analytical classifications, descriptive distributions, worked examples and construct refinements.

## Declarations

### Use of generative artificial intelligence

Generative AI assisted the preparation of files and manuscript editing. Analytical decisions remained the responsibility of the researchers. Source verification, independent analytical review and resolution of disagreements followed the procedures described in the Methods and were documented in the analysis workbook.

## References

[1] World Health Organization. WHO global strategy on people-centred and integrated health services: interim report. No. WHO/HIS/SDS/2015.6. World Health Organization; 2015.

[2] Teisberg E, Wallace S, O’Hara S. Defining and Implementing Value-Based Health Care: A Strategic Framework. Acad Med. 2020;95(5):682–5. 10.1097/ACM.0000000000003122. PMC7185050 31833857

[3] Garcia J, Reddy CL, Atun R. Transitioning to High-Value Health Systems in G20 + Countries. Harvard University; 2023. 10.54111/0001/HSIL/thvhs.

[4] Porter ME. What Is Value in Health Care? N Engl J Med. 2010;363(26):2477–81. 10.1056/NEJMp1011024. 21142528

[5] Greenhalgh T, Robert G, Macfarlane F, Bate P, Kyriakidou O. Diffusion of Innovations in Service Organizations: Systematic Review and Recommendations. Milbank Q. 2004;82(4):581–629. 10.1111/j.0887-378X.2004.00325.x. PMC2690184 15595944

[6] Ferlie EB, Shortell SM. Improving the Quality of Health Care in the United Kingdom and the United States: A Framework for Change. Milbank Q. 2001;79(2):281–315. 10.1111/1468-0009.00206. PMC2751188 11439467

[7] Bossert T. Analyzing the decentralization of health systems in developing countries: decision space, innovation and performance. Soc Sci Med. 1998;47(10):1513-27. 10.1016/s0277-9536(98)00234-2. PMID: 9823047. 9823047

[8] Bossert TJ, Mitchell AD. Health sector decentralization and local decision-making: Decision space, institutional capacities and accountability in Pakistan. Soc Sci Med. 2011;72(1):39–48. 10.1016/j.socscimed.2010.10.019. 21134705

[9] Mintzberg H. Understanding Organizations… Finally! Structuring in Sevens. Oakland,CA: Berrett-Koehler Publishers; 2023.

[10] Gittell J, Hoffer. High performance healthcare: Using the power of relationships to achieve quality, efficiency and resilience. McGraw-Hill; 2009.

[11] Adler PS, Borys B. Two types of bureaucracy. Adm Sci Q. 1996;41(1):61–89. 10.2307/2393986.

[12] Khalil H, Ameen M, Davies C, Liu C. Implementing value-based healthcare: a scoping review of key elements, outcomes, and challenges for sustainable healthcare systems. Front public health. 2025;13:1514098. 10.3389/fpubh.2025.1514098. PMC12014573 40270723

[13] Damberg CL, Sorbero ME, Lovejoy SL, Martsolf GR, Raaen L, Mandel D. Measuring Success in Health Care Value-Based Purchasing Programs: Findings from an Environmental Scan, Literature Review, and Expert Panel Discussions. Rand Health Q. 2014;4(3):9. PMID: 28083347; PMCID: PMC5161317. 10.7249/rhq-04-03-09 PMC5161317 28083347

[14] Grozdanova A, Mihajloska E, Naumovska Z, Staninova Stojovska M, Sterjev Z, Dimkovski A, et al. Toward value in healthcare: opportunities and challenges for implementing Value-Based Healthcare in North Macedonia. Front public health. 2025;13:1646870. 10.3389/fpubh.2025.1646870. PMC12568455 41170483

[15] Van Herck P, De Smedt D, Annemans L, Remmen R, Rosenthal MB, Sermeus W. Systematic review: Effects, design choices, and context of pay-for-performance in health care. BMC Health Serv Res. 2010;10(1). 10.1186/1472-6963-10-247. PMC2936378 20731816

[16] Evans PB. Embedded Autonomy: States and Industrial Transformation. Princeton, NJ: Princeton University Press; 1995.

[17] Ohrling M, Tolf S, Solberg Carlsson K, Brommels M. That’s how it should work: the perceptions of senior management on the value of decentralization. J Health Organ Manag. 2021b;35(5):596–613. 10.1108/JHOM-12-2020-0474 33905183

[18] Ohrling M, Tolf S, Solberg-Carlsson K, Brommels M. Managers do it their way: how managers act in a decentralized healthcare services provider organization. Health Serv Manage Res. 2022a;35(4):215–28. 10.1177/09514848211065467 34963355

[19] Ohrling M, Solberg Carlsson K, Brommels M. No man is an island: management of the emergency response to the SARS-CoV-2 (COVID-19) outbreak in a large public decentralised service delivery organisation. BMC health Serv Res March 21, 2022b;22(1). 10.1186/s12913-022-07716-w PMC8935606 35313891

[20] Ohrling M. Challenges in hybrid management in healthcare: a study of the interplay between divisional managers and clinical directors in a decentralized healthcare organization in Sweden. BMC Health Serv Res. 2026;26(1). 10.1186/s12913-025-13977-y. PMC12849398 41526882

[21] Ohrling M, Øvretveit J, Brommels M. Can management decentralisation resolve challenges faced by healthcare service delivery organisations? Findings for managers and researchers from a scoping review. Int J Health Plann Manag. 2021a;36(1):30–41. 10.1002/hpm.3058. 32862493

[22] Orlikowski WJ. The duality of technology: Rethinking the concept of technology in organizations. Organ Sci. 1992;3(3):398–427. 10.1287/orsc.3.3.398.

[23] Ohrling M. Having and eating the cake? The centralisation paradox in decentralised healthcare governance: a scoping review. BMC Health Serv Res. 2026;26:1020. 10.1186/s12913-026-15162-1. PMC13397811 42498925

[24] Suddaby R. Editor’s comments: construct clarity in theories of management and organization. Acad Manag Rev. 2010;35(3):346–57. 10.5465/amr.35.3.zok346.

[25] Whetten DA. What constitutes a theoretical contribution? Acad Manag Rev. 1989;14(4):490–5. 10.5465/amr.1989.4308371.

[26] Jabareen Y. Building a Conceptual Framework: Philosophy, Definitions, and Procedure. Int J qualitative methods. 2009;8(4):49–62. 10.1177/160940690900800406.

[27] Peters MDJ, Marnie C, Tricco AC, Pollock D, Munn Z, Alexander L, McInerney P, Godfrey CM, Khalil H. Updated methodological guidance for the conduct of scoping reviews. JBI Evid Synth. 2020;18(10):2119–26. 10.11124/JBIES-20-00167. 33038124

[28] Carroll C, Booth A, Leaviss J, Rick J. Best fit framework synthesis: refining the method. BMC Med Res Methodol. 2013;13:37. 10.1186/1471-2288-13-37. PMC3618126 23497061

[29] Hsieh HF, Shannon SE. Three approaches to qualitative content analysis. Qual Health Res. 2005;15(9):1277–88. 10.1177/1049732305276687. 16204405

[30] Tavory I, Timmermans S. Abductive analysis: theorizing qualitative research. Chicago: University of Chicago Press; 2014.

[31] Arksey H, O’Malley L. Scoping studies: towards a methodological framework. Int J Soc Res Methodol. 2005;8(1):19–32.

[32] Munn Z, Peters MDJ, Stern C, Tufanaru C, McArthur A, Aromataris E. Systematic review or scoping review? Guidance for authors when choosing between a systematic or scoping review approach. BMC Med Res Methodol. 2018;18:143. 10.1186/s12874-018-0611-x. PMC6245623 30453902

[33] Tricco AC, Lillie E, Zarin W, et al. PRISMA extension for scoping reviews (PRISMA-ScR): checklist and explanation. Ann Intern Med. 2018;169(7):467–73. 10.7326/M18-0850. 30178033

[34] Rethlefsen ML, Kirtley S, Waffenschmidt S, Ayala AP, Moher D, Page MJ, Koffel JB, PRISMA-S Group. PRISMA-S: an extension to the PRISMA Statement for Reporting Literature Searches in Systematic Reviews. Syst Rev. 2021;10:39. 10.1186/s13643-020-01542-z. PMC8270366 34285662

[35] Alawode GB, Ajibola AA, Afolabi OR, Nkwain C, Alawode KA. An enabler or a barrier: implications of local government autonomy for effective primary health care (PHC) reforms in Nigeria. Pan Afr Med J. 2026;53:30. 10.11604/pamj.2026.53.30.49847. PMC13156014 42111507

[36] Allawati NR, Mohamed N, Al Nadabi W. An evaluation of three decades of health system decentralisation in Oman. East Mediterr Health J. 2026;32(1):12–8. 10.26719/2026.32.1.12. 41761886

[37] Amollo NW, Ogol J, Museve E, Owenga JA, Aduda DO, Onguru D. Financial determinants of effective hypertension and diabetes care in rural primary health facilities in Kisumu, Kenya: a mixed-methods study. BMC Public Health. 2026;26(1):1275. 10.1186/s12889-026-26963-8. PMC13094046 41814241

[38] Atkinson S, Medeiros RL, Oliveira PH, de Almeida RD. Going down to the local: incorporating social organisation and political culture into assessments of decentralised health care. Soc Sci Med. 2000;51(4):619–36. 10.1016/s0277-9536(00)00005-8. 10868675

[39] Ayim A, Agyepong IA, Enyimayew N. Why district assemblies disburse resources to district health systems for service delivery at district level in the context of decentralization: a comparative study of two districts in the Volta Region of Ghana. Front Public Health. 2023;11:1136210. 10.3389/fpubh.2023.1136210. PMC10461447 37645704

[40] Baird A, Furukawa MF, Rahman B, Schneller ES. Corporate governance and the adoption of health information technology within integrated delivery systems. Health Care Manage Rev. 2014;39(3):234–44. 10.1097/hmr.0b013e318294e5e6. 23624831

[41] Barasa EW, Molyneux S, English M, Cleary S. Hospitals as complex adaptive systems: a case study of factors influencing priority setting practices at the hospital level in Kenya. Soc Sci Med. 2017;174:104–12. 10.1016/j.socscimed.2016.12.026. PMC5267634 28024239

[42] Bernardes A, Cummings G, Évora YD, Gabriel CS. Framing the difficulties resulting from implementing a participatory management model in a public hospital. Rev Lat Am Enfermagem. 2012;20(6):1142–51. 10.1590/s0104-11692012000600017. 23258728

[43] Binyaruka P, Maiba J, Mshana D, Gatome-Munyua A, Mtei G. Does provider autonomy work well in Tanzania? Perspectives of primary care facilities on budget execution under direct facility financing and factors affecting provider autonomy in Singida region. Health Syst Reform. 2024;10(3):2432043. 10.1080/23288604.2024.2432043. 39652153

[44] Birru E, Ndayizigiye M, Wanje G, Marole T, Smith PD, Koto M, et al. Healthcare workers’ views on decentralized primary health care management in Lesotho: a qualitative study. BMC Health Serv Res. 2024;24(1):801. 10.1186/s12913-024-11279-3. PMC11241925 38992665

[45] Björk L, Corin L, Akerstrom M, Jonsdottir IH, Innocenti AD, Wijk H, et al. Under pressure - the working situation of Swedish healthcare managers during the first wave of COVID-19. Front Psychol. 2023;13:1052382. 10.3389/fpsyg.2022.1052382. PMC9874142 36710753

[46] Bossert TJ, Mitchell AD, Janjua MA. Improving health system performance in a decentralized health system: capacity building in Pakistan. Health Syst Reform. 2015;1(4):276–84. 10.1080/23288604.2015.1056330. 31519095

[47] Büchner VA, Schreyögg J, Schultz C. The impact of the board’s strategy-setting role on board-management relations and hospital performance. Health Care Manage Rev. 2014;39(4):305–17. 10.1097/hmr.0b013e31829fca10. 23896658

[48] Chama-Chiliba CM, Hangoma P, Chansa C, Mulenga MC. Effects of performance based financing on facility autonomy and accountability: evidence from Zambia. Health Policy Open. 2022;3:100061. 10.1016/j.hpopen.2021.100061. PMC10297809 37383569

[49] Conn CP, Jenkins P, Touray SO. Strengthening health management: experience of district teams in The Gambia. Health Policy Plan. 1996;11(1):64–71. 10.1093/heapol/11.1.64. 10155879

[50] Do MH, Bui TTH, Phan T, Nguyen HL, Duong TA, Le BC, et al. Strengthening public health management capacity in Vietnam: preparing local public health workers for new roles in a decentralized health system. J Public Health Manag Pract. 2018;24(Suppl 2):S74–81. 10.1097/phh.0000000000000755. 29369260

[51] Dubé A, Collin S, Hakim J, Johnson C, Laforest ME, Landry MH, et al. From policy to practice: a qualitative study on reforms and frontline retention in healthcare. Inquiry. 2025;62:469580251365821. 10.1177/00469580251365821. PMC12357990 40817824

[52] Duran A, Chanturidze T, Gheorghe A, Moreno A. Assessment of public hospital governance in Romania: lessons from 10 case studies. Int J Health Policy Manag. 2019;8(4):199–210. 10.15171/ijhpm.2018.120. PMC6499904 31050965

[53] Erchick DJ, George AS, Umeh C, Wonodi C. Understanding internal accountability in Nigeria’s routine immunization system: perspectives from government officials at the national, state, and local levels. Int J Health Policy Manag. 2017;6(7):403–12. 10.15171/ijhpm.2016.150. PMC5505110 28812836

[54] Feldhaus I, Chatterjee S, Clarke-Deelder E, Brenzel L, Resch S, Bossert TJ. Examining decentralization and managerial decision making for child immunization program performance in India. Soc Sci Med. 2023;317:115457. 10.1016/j.socscimed.2022.115457. PMC9870749 36493499

[55] Geng J, Yu H, Chen Y. Preparing for the introduction of hospital autonomy in Laos: an assessment of current situation and suggestions for policy-making. Int J Health Plann Manage. 2016;31(2):148–66. 10.1002/hpm.2283. 25677738

[56] George AS, Erchick DJ, Zubairu MM, Barau IY, Wonodi C. Sparking, supporting and steering change: grounding an accountability framework with viewpoints from Nigerian routine immunization and primary health care government officials. Health Policy Plan. 2016;31(9):1326–32. 10.1093/heapol/czw057. 27198980

[57] Gobezie DA, Gebeyehu EM, Yasine T, Abeje ET, Mekonen AM. Healthcare governance practices and their determinants among public hospital managers in South Wollo zone, Northeast Ethiopia. Sci Rep. 2025;15(1):33984. 10.1038/s41598-025-12134-z. PMC12484764 41028140

[58] Grafton J, Abernethy MA, Lillis AM. Organisational design choices in response to public sector reforms: a case study of mandated hospital networks. Manag Acc Res. 2011;22(4):242–68. 10.1016/j.mar.2011.06.001.

[59] Heerdegen ACS, Gerold J, Amon S, Agyemang SA, Aikins M, Wyss K. How does district health management emerge within a complex health system? Insights for capacity strengthening in Ghana. Front Public Health. 2020;8:270. 10.3389/fpubh.2020.00270. PMC7360672 32733833

[60] Henriksson DK, Ayebare F, Waiswa P, Peterson SS, Tumushabe EK, Fredriksson M. Enablers and barriers to evidence based planning in the district health system in Uganda; perceptions of district health managers. BMC Health Serv Res. 2017;17(1):103. 10.1186/s12913-017-2059-9. PMC5289024 28148251

[61] Islam M. The influence of politics on accountability of health professionals in Bangladesh: an analysis of the quality of health service delivery. Asia Pac J Health Manag. 2017;12(1):75–82. 10.24083/apjhm.v12i1.101.

[62] Jack W. Contracting for health services: an evaluation of recent reforms in Nicaragua. Health Policy Plan. 2003;18(2):195–204. 10.1093/heapol/czg024. 12740324

[63] Jalilvand MA, Raeisi AR, Shaarbafchizadeh N. Hospital governance accountability challenges in Iran: a qualitative study. BMC Health Serv Res. 2025;25(1):946. 10.1186/s12913-025-13100-1. PMC12243261 40640820

[64] Kawonga M, Fonn S, Blaauw D. Administrative integration of vertical HIV monitoring and evaluation into health systems: a case study from South Africa. Glob Health Action. 2013;6:19252. 10.3402/gha.v6i0.19252. PMC3556718 23364092

[65] Kesale AM, Mahonge C, Muhanga M. The quest for accountability of health facility governing committees implementing direct health facility financing in Tanzania: a supply-side experience. PLoS ONE. 2022;17(4):e0267708. 10.1371/journal.pone.0267708. PMC9049541 35482793

[66] Kielmann K, Dickson-Hall L, Jassat W, Le Roux S, Moshabela M, Cox H, et al. We had to manage what we had on hand, in whatever way we could’: adaptive responses in policy for decentralized drug-resistant tuberculosis care in South Africa. Health Policy Plan. 2021;36(3):249–59. 10.1093/heapol/czaa147. PMC8059133 33582787

[67] Kigume R, Maluka S. Health sector decentralisation in Tanzania: how do institutional capacities influence use of decision space? Int J Health Plann Manage. 2018;33(4):e1050–66. 10.1002/hpm.2587. 30052278

[68] Kigume R, Maluka S. Decentralisation and health services delivery in 4 districts in Tanzania: how and why does the use of decision space vary across districts? Int J Health Policy Manag. 2019;8(2):90–100. 10.15171/ijhpm.2018.97. PMC6462210 30980622

[69] Kigume R, Maluka S. Health sector decentralisation in Tanzania: analysis of decision space in human resources for health management. Int J Health Plann Manage. 2019;34(4):1265–76. 10.1002/hpm.2792. 30994219

[70] Kigume R, Maluka S, Kamuzora P. Decentralisation and health services delivery in Tanzania: analysis of decision space in planning, allocation, and use of financial resources. Int J Health Plann Manage. 2018;33(2):e621–35. 10.1002/hpm.2511. 29542196

[71] Kuwawenaruwa A, Remme M, Mtei G, Makawia S, Maluka S, Kapologwe N, et al. Bank accounts for public primary health care facilities: reflections on implementation from three districts in Tanzania. Int J Health Plann Manage. 2019;34(1):e860–74. 10.1002/hpm.2702. 30461049

[72] Kwamie A, Agyepong IA, van Dijk H. What governs district manager decision making? A case study of complex leadership in Dangme West District, Ghana. Health Syst Reform. 2015;1(2):167–77. 10.1080/23288604.2015.1032475. 31546310

[73] Kwamie A, van Dijk H, Ansah EK, Agyepong IA. The path dependence of district manager decision-space in Ghana. Health Policy Plan. 2016;31(3):356–66. 10.1093/heapol/czv069. PMC4779147 26318537

[74] Lewis R, Hinton L. Citizen and staff involvement in health service decision-making: have National Health Service foundation trusts in England given stakeholders a louder voice? J Health Serv Res Policy. 2008;13(1):19–25. 10.1258/jhsrp.2007.007003. 18325152

[75] Liwanag HJ, Wyss K. What conditions enable decentralization to improve the health system? Qualitative analysis of perspectives on decision space after 25 years of devolution in the Philippines. PLoS ONE. 2018;13(11):e0206809. 10.1371/journal.pone.0206809. PMC6218067 30395625

[76] Liwanag HJ, Wyss K. Optimising decentralisation for the health sector by exploring the synergy of decision space, capacity and accountability: insights from the Philippines. Health Res Policy Syst. 2019;17(1):4. 10.1186/s12961-018-0402-1. PMC6327786 30630469

[77] Liwanag HJ, Wyss K. Who should decide for local health services? A mixed methods study of preferences for decision-making in the decentralized Philippine health system. BMC Health Serv Res. 2020;20(1):305. 10.1186/s12913-020-05174-w. PMC7158124 32293432

[78] Malcolm L, Alp B, Bryson J. Decentralisation of general management within the New Zealand health system. Health Serv Manage Res. 1994;7(4):220–8. 10.1177/095148489400700402. 10138683

[79] Malcolm L, Barnett P. Decentralisation, integration and accountability: perceptions of New Zealand’s top health service managers. Health Serv Manage Res. 1995;8(2):121–34. 10.1177/095148489500800204. 10143979

[80] Maluka S, Kamuzora P, San Sebastiån M, Byskov J, Olsen ØE, Shayo E, et al. Decentralized health care priority-setting in Tanzania: evaluating against the accountability for reasonableness framework. Soc Sci Med. 2010;71(4):751–9. 10.1016/j.socscimed.2010.04.035. 20554365

[81] Maluka S, Kamuzora P, Sansebastián M, Byskov J, Ndawi B, Olsen ØE, et al. Implementing accountability for reasonableness framework at district level in Tanzania: a realist evaluation. Implement Sci. 2011;6:11. 10.1186/1748-5908-6-11. PMC3041695 21310021

[82] Marchal B, Dedzo M, Kegels G. A realist evaluation of the management of a well-performing regional hospital in Ghana. BMC Health Serv Res. 2010;10:24. 10.1186/1472-6963-10-24. PMC2828434 20100330

[83] McCollum R, Theobald S, Otiso L, Martineau T, Karuga R, Barasa E, et al. Priority setting for health in the context of devolution in Kenya: implications for health equity and community-based primary care. Health Policy Plan. 2018;33(6):729–42. 10.1093/heapol/czy043. PMC6005116 29846599

[84] McDermott AM, Fitzgerald L, Van Gestel NM, Keating MA. From bipartite to tripartite devolved HRM in professional service contexts: evidence from hospitals in three countries. Hum Resour Manage. 2015;54(5):813–31. 10.1002/hrm.21728.

[85] McPake B, Yepes FJ, Lake S, Sanchez LH. Is the Colombian health system reform improving the performance of public hospitals in Bogotá? Health Policy Plan. 2003;18(2):182–94. 10.1093/heapol/czg023. 12740323

[86] Mkoka DA, Kiwara A, Goicolea I, Hurtig AK. Governing the implementation of emergency obstetric care: experiences of rural district health managers, Tanzania. BMC Health Serv Res. 2014;14:333. 10.1186/1472-6963-14-333. PMC4124475 25086597

[87] Mohammed J, North N, Ashton T. Decentralisation of health services in Fiji: a decision space analysis. Int J Health Policy Manag. 2016;5(3):173–81. 10.15171/ijhpm.2015.199. PMC4770923 26927588

[88] Munywoki J, Kagwanja N, Chuma J, Nzinga J, Barasa E, Tsofa B. Tracking health sector priority setting processes and outcomes for human resources for health, five-years after political devolution: a county-level case study in Kenya. Int J Equity Health. 2020;19(1):165. 10.1186/s12939-020-01284-3. PMC7507677 32958000

[89] Mutemwa RI. HMIS and decision-making in Zambia: re-thinking information solutions for district health management in decentralized health systems. Health Policy Plan. 2006;21(1):40–52. 10.1093/heapol/czj003. 16319088

[90] Newbrander W, Peercy C, Shepherd-Banigan M, Vergeer P. A tool for assessing management capacity at the decentralized level in a fragile state. Int J Health Plann Manage. 2012;27(4):276–94. 10.1002/hpm.1108. 22034286

[91] Nxumalo N, Gilson L, Goudge J, Tsofa B, Cleary S, Barasa E, et al. Accountability mechanisms and the value of relationships: experiences of front-line managers at subnational level in Kenya and South Africa. BMJ Glob Health. 2018;3(4):e000842. 10.1136/bmjgh-2018-000842. PMC6038841 30002921

[92] Nyikuri M, Tsofa B, Barasa E, Okoth P, Molyneux S. Crises and resilience at the frontline-public health facility managers under devolution in a sub-county on the Kenyan coast. PLoS ONE. 2015;10(12):e0144768. 10.1371/journal.pone.0144768. PMC4687913 26696096

[93] Nyikuri MM, Tsofa B, Okoth P, Barasa EW, Molyneux S. We are toothless and hanging, but optimistic: sub county managers’ experiences of rapid devolution in coastal Kenya. Int J Equity Health. 2017;16(1):113. 10.1186/s12939-017-0607-x. PMC5599878 28911332

[94] Nyland K, Pettersen IJ. Hybrid controls and accountabilities in public sector management: three case studies in a reforming hospital sector. Int J Public Sect Manag. 2015;28(2):90–104. 10.1108/ijpsm-07-2014-0085.

[95] Panda B, Zodpey SP, Thakur HP. Local self governance in health - a study of it’s functioning in Odisha, India. BMC Health Serv Res. 2016;16(Suppl 6):554. 10.1186/s12913-016-1785-8. PMC5103239 28185587

[96] Qazi MS, Ali M. Pakistan’s health management information system: health managers’ perspectives. J Pak Med Assoc. 2009;59(1):10–4. PMID: 19213369. 19213369

[97] Rodríguez DC, Balaji LN, Chamdimba E, Kafumba J, Koon AD, Mazalale J, et al. Political economy analysis of subnational health management in Kenya, Malawi and Uganda. Health Policy Plan. 2023;38(5):631–47. 10.1093/heapol/czad021. PMC10190959 37084282

[98] Saxena A, Davies M, Philippon D. Structure of health-care dyad leadership: an organization’s experience. Leadersh Health Serv (Bradf Engl). 2018;31(2):238–53. 10.1108/lhs-12-2017-0076. 29771222

[99] Shaikh S, Naeem I, Nafees A, Zahidie A, Fatmi Z, Kazi A. Experience of devolution in district health system of Pakistan: perspectives regarding needed reforms. J Pak Med Assoc. 2012;62(1):28–32. PMID: 22352097. 22352097

[100] Shayo EH, Mboera LE, Blystad A. Stakeholders’ participation in planning and priority setting in the context of a decentralised health care system: the case of prevention of mother to child transmission of HIV programme in Tanzania. BMC Health Serv Res. 2013;13:273. 10.1186/1472-6963-13-273. PMC3720200 23849730

[101] Ssengooba F, Atuyambe L, McPake B, Hanson K, Okuonzi S. What could be achieved with greater public hospital autonomy? Comparison of public and PNFP hospitals in Uganda. Public Adm Dev. 2002;22(5):415–28. 10.1002/pad.244.

[102] Storey J, Holti R. Sense-making by clinical and non-clinical executive directors within new governance arrangements. J Health Organ Manag. 2009;23(2):149–69. 10.1108/14777260910960902. 19711775

[103] Sucaldito NL, Tayag EA, Roces MC, Malison MD, Robie BD, Howze EH. The Philippines field management training program (FMTP): strengthening management capacity in a decentralized public health system. Int J Public Health. 2014;59(6):897–903. 10.1007/s00038-014-0603-5. 25238870

[104] Sumah AM, Baatiema L. Decentralisation and management of human resource for health in the health system of Ghana: a decision space analysis. Int J Health Policy Manag. 2019;8(1):28–39. 10.15171/ijhpm.2018.88. PMC6358646 30709100

[105] Svensson I. Taking sides or bridging worlds? Managerial responses to conflicts and tensions between the core operations and the administration in healthcare. BMC Health Serv Res. 2025;25(1):1453. 10.1186/s12913-025-13659-9. PMC12595710 41206441

[106] Thongngun T, Tiraphat S. Provincial lessons on long-term care implementation in Thailand: motivation and managerial capacities within a decentralized health system in a middle-income country. BMC Health Serv Res. 2026;26(1):778. 10.1186/s12913-026-14571-6. PMC13224624 42001105

[107] Tsofa B, Goodman C, Gilson L, Molyneux S. Devolution and its effects on health workforce and commodities management - early implementation experiences in Kilifi County, Kenya. Int J Equity Health. 2017;16(1):169. 10.1186/s12939-017-0663-2. PMC5599882 28911328

[108] Tsofa B, Molyneux S, Gilson L, Goodman C. How does decentralisation affect health sector planning and financial management? A case study of early effects of devolution in Kilifi County, Kenya. Int J Equity Health. 2017;16(1):151. 10.1186/s12939-017-0649-0. PMC5599897 28911325

[109] Tsofa B, Waweru E, Munywoki J, Soe K, Rodriguez DC, Koon AD. Political economy analysis of sub-national health sector planning and budgeting: a case study of three counties in Kenya. PLOS Glob Public Health. 2023;3(1):e0001401. 10.1371/journal.pgph.0001401. PMC10022076 36962920

[110] Waweru E, Goodman C, Kedenge S, Tsofa B, Molyneux S. Tracking implementation and (un)intended consequences: a process evaluation of an innovative peripheral health facility financing mechanism in Kenya. Health Policy Plan. 2016;31(2):137–47. 10.1093/heapol/czv030. PMC4748125 25920355

[111] Witter S, van der Merwe M, Twine R, Mabetha D, Hove J, Tollman SM, et al. Opening decision spaces: a case study on the opportunities and constraints in the public health sector of Mpumalanga province, South Africa. PLoS ONE. 2024;19(7):e0304775. 10.1371/journal.pone.0304775. PMC11226100 38968289

[112] Power M. The audit society: rituals of verification. Oxford: Oxford Univ. Press; 2000.

[113] Hood C, Dixon R. Not What It Said on the Tin? Reflections on Three Decades of UK Public Management Reform. Financial Account Manage. 2016;32(4):409–28. 10.1111/faam.12095.

[114] Bevan G, Hood C. What’s measured is what matters: targets and gaming in the english public health care system. Public Adm (London) August. 2006;84(3):517–38. 10.1111/j.1467-9299.2006.00600.x.

[115] Dal Mas F, Massaro M, Rippa P, Secundo G. The challenges of digital transformation in healthcare: an interdisciplinary literature review, framework, and future research agenda. Technovation. 2023;123. 10.1016/j.technovation.2023.102716.

[116] Cresswell K, Sheikh A. Organizational issues in the implementation and adoption of health information technology innovations: An interpretative review. Int J Med Inf (Shannon Ireland). 2013;82(5):e73–86. 10.1016/j.ijmedinf.2012.10.007. 23146626

[117] Lair CD. Outsourcing and the Risks of Dependent Autonomy. SAGE open. 2019;9(2). 10.1177/2158244019845177.

[118] Mackenzie C, Stoljar N, editors. Relational autonomy: feminist perspectives on autonomy, agency, and the social self. New York: Oxford University Press; 2000.

[119] Ansell C, Sørensen E, Torfing J. Public administration and politics meet turbulence: The search for robust governance responses. Public Adm (London). 2023;101(1):3–22. 10.1111/padm.12874.

[120] Filgueiras F, Palotti P, Testa GG. Complexing Governance Styles: Connecting Politics and Policy in Governance Theories. SAGE open. 2023;13(1). 10.1177/21582440231158521.

