# Supplementary material for: Dependent autonomy: developing and refining a theoretical construct of managerial autonomy in healthcare
Source: BMC Health Serv Res. 2026 Nov 9;26:1561. doi: 10.1186/s12913-026-16096-4 (PMC13749796; doi:10.1186/s12913-026-16096-4)
Supplement: Supplementary file 1 [file 12913_2026_16096_MOESM1_ESM.pdf]

Supplementary File 1

Stage 1 analytical procedure and traceability

This supplement explains the development of the seven Stage 1 coding categories, illustrates their contribution to the three themes and provisional construct of dependent autonomy, and maps their transition into the Stage 2 domains. Worked examples draw on the four empirical programme studies [17-20]. The preceding scoping review [21] provided conceptual orientation. A separate descriptive inventory preserves the original coding record across all five publications; its counts are not counts of eligible empirical findings.

S1 Development of the coding categories

The initial categories were developed deductively using directed qualitative content analysis [29]. Decision Space Theory informed distinctions between formal authority, its practical exercise, capacity and accountability [7,8]. Organisational theory informed attention to enabling and constraining systems [11]. The seven category labels and coding boundaries were formulated for this study; they are not a prescribed taxonomy from these theories.

Table S1.1. Theoretical basis and scope of the seven analytical categories.

Analytical category	Theoretical basis and coding focus	Theme
Formal decision authority	Decision Space Theory [7,8]: formally delegated decision rights and responsibilities.	T1
Enacted decision space	Decision Space Theory [7,8]: reported use or non-use of delegated authority in managerial practice.	T1
Bounded autonomy	Decision Space Theory [7,8]: limits, contingencies and discrepancies between formal authority and practical discretion.	T1
Support systems	Capacity [8] and enabling or constraining systems [11]: administrative and professional expertise, resources and managerial development.	T2
Information infrastructure	Capacity [8] and organisational systems [11]: information, feedback and digital arrangements relevant to interpretation and action.	T2
Central coordination	Decentralisation and coordination [21], interpreted through organisational systems [11]: arrangements connecting units and managerial levels.	T3
Role ambiguity	Accountability [8]: clarity or uncertainty concerning responsibility, decision ownership and who is entitled to act.	T3

Theme key

T1 Enabled yet contingent autonomy
T2 Organisational capacity as a conditioning force
T3 Coordination as integrative yet complexity-generating

The categories organised the statements. The themes were developed through comparison across categories and studies, followed by theoretical interpretation.

S2 Descriptive inventory of the original coding record

The original coding inventory contains 220 recorded entries: 178 from the four empirical publications and 42 from the preceding scoping review. Table S1.2 shows their source distribution; Table S1.3 shows their allocation to categories and themes. The review entries document its sensitising role and are excluded from the subtotal for the empirical publications.

Table S1.2. Recorded entries by source publication in the original coding inventory.

Source publication	Recorded entries, n	Analytical role
Senior-management programme theory (2021) [17]	41	Empirical study
Clinical directors' decision space (2022) [18]	43	Empirical study
Crisis management (2022) [19]	50	Empirical study
Hybrid managerial arrangements (2026) [20]	44	Empirical study
**Subtotal: four empirical publications**	**178**	Entries from empirical publications
Preceding scoping review (2021) [21]	42	Sensitising source; excluded from empirical subtotal
**Total: original programme coding inventory**	**220**	**Complete inventory**

Note. Recorded entries include empirical observations alongside contextual, methodological and interpretive material, with some overlapping observations. Neither the total nor the empirical-publication subtotal is a verified count of eligible analytical evidence statements. Counts do not indicate prevalence, independent observations, evidential strength or conceptual importance.

Table S1.3. Category and theme distribution in the original coding inventory.

Theme	Analytical category	Recorded entries, n	Theme total, n
Enabled yet contingent autonomy	Formal decision authority	25	86
	Enacted decision space	28	
	Bounded autonomy	33	
Organisational capacity as a conditioning force	Support systems	33	65
	Information infrastructure	32	
Coordination as integrative yet complexity-generating	Central coordination	34	69
	Role ambiguity	35	
**Total**		**220**	**220**

Note. Theme totals include the 42 entries from the preceding scoping review [21]. They do not represent the distribution of eligible empirical findings from the four Stage 1 studies [17-20].

S3 Worked examples

The following selected examples illustrate condensation and coding. Evidence statements are paraphrases of published findings.

Example 1 Distinguishing formal authority from practical discretion

[18], Results, “Decision space”, PDF p. 6. Published excerpt:

“formally granted large decision space, but the actual decision space was perceived as more limited.”

Statement: formal delegation granted broad authority, but managers perceived their practical scope for action as narrower. Coding: bounded autonomy (T1), because the dominant meaning is the discrepancy between formal authority and practical discretion.

Example 2 Identifying organisational support for managerial action

[19], Results, “SLSO’s support system was mostly available when needed”, PDF p. 12. Published excerpt:

“Examples cited were ordering PPE, additional transportation, support for treatment, help from human resources regarding rostering, and legal advice regarding staff rights.”

Statement: managers reported access to operational assistance, HR expertise and legal advice during the pandemic response. Coding: support systems (T2), because the passage identifies concrete support for carrying out managerial responsibilities.

Example 3 Separating two organisational conditions

[20], Results, “Objective 2: Reduced administrative burden”, PDF p. 5. Published excerpt:

“Respondents frequently mentioned unclear responsibilities and lack of digital support as contributing factors.”

Statement 1: respondents linked unclear responsibilities to continuing administrative burden. Coding: role ambiguity (T3). Statement 2: respondents linked insufficient digital support to continuing administrative burden. Coding: information infrastructure (T2).

The two separable conditions receive distinct statements and categories but remain linked to the same source passage. They are not independent observations.

For ambiguous passages, coding followed the dominant meaning. For example, uncertainty about who owned decisions communicated during the pandemic [19, PDF p. 12] was coded as role ambiguity, although the passage also concerned information.

S4 From comparison to themes and the provisional construct

These comparisons illustrate how relationships across findings informed thematic abstraction. They retain both enabling conditions and qualifications within the programme evidence.

T1 Enabled yet contingent autonomy

Senior managers described delegation of responsibility for budgets, staffing, care quality and work environment [17, PDF p. 8]. Clinical directors reported using delegated authority, while also describing narrower practical discretion [18, PDF pp. 6–7]. Comparing formal mandates with reported use and constraints connected the categories formal decision authority, enacted decision space and bounded autonomy. The theme captures authority that can be exercised but remains conditional on organisational circumstances.

T2 Organisational capacity as a conditioning force

Managers described useful operational, HR and legal support during the pandemic [19, PDF p. 12]. The hybrid-management study reported improved access to expertise in some divisions alongside inconsistent support and gaps in digital provision [20, PDF pp. 5–6]. Comparison brought support systems and information infrastructure together around the availability and practical usability of organisational capacity.

T3 Coordination as integrative yet complexity generating

Managers described geographic clusters as supporting staffing, information sharing and collaboration during the pandemic, alongside barriers to sustaining collaboration [19, PDF p. 11]. Following the introduction of a divisional level, respondents reported improved coordination but linked unclear responsibilities to continuing administrative burden [20, PDF p. 5]. Comparing these findings linked central coordination and role ambiguity in the theme coordination as integrative yet complexity-generating.

Contribution to construct generation

Together, the themes informed dependent autonomy as a proposed theoretical construct describing the organisationally conditioned actionability of formally delegated managerial authority. T1 identifies the relationship between formal authority and its practical exercise. T2 and T3 specify organisational conditions that may enable or constrain that exercise. This provisional formulation provided the starting point for the separate external review in Stage 2.

Analytical responsibility and traceability

The author (MO) conducted extraction, condensation, coding, comparison and conceptual synthesis. Coding decisions and revisions were documented in an audit trail. Two senior researchers critically reviewed the provisional construct but did not independently code the material. The examples illustrate interpretive links and do not establish prevalence, independent replication or causal effects.

Source locators

Reference numbers follow the main manuscript. PDF page numbers count from the first page of the source file.

[17] That’s how it should work (2021). Activities based on the underlying assumptions. doi:10.1108/JHOM-12-2020-0474.

[18] Managers do it their way (2022). Results and Decision space. doi:10.1177/09514848211065467.

[19] No man is an island (2022). Results. doi:10.1186/s12913-022-07716-w.

[20] Challenges in hybrid management in healthcare (2026). Results, Objectives 2 and 3. doi:10.1186/s12913-025-13977-y.

S5 Carrying Stage 1 categories into Stage 2

The seven theory-informed categories structured coding in Stage 1. Comparison across the programme studies generated three overarching themes and informed the provisional construct of dependent autonomy. The same categories were then carried forward as provisional organising domains for Stage 2 (Table S1.4). They were not seven new empirical findings generated after thematic abstraction.

In Stage 2, a domain denotes a broad area used to organise external findings. Some category labels were broadened to encompass enabling and constraining conditions across healthcare contexts. This change in wording preserved the connection to Stage 1 while allowing a wider scope for external comparison.

Table S1.4. Mapping of Stage 1 analytical categories to provisional Stage 2 domains and rationale for their formulation.

Stage 1 analytical category	Provisional Stage 2 domain	Underlying organisational question	Basis for continuity or broadening
**Formal decision authority**	Formal decision authority	What decisions are managers formally authorised and accountable to make?	Label retained to identify the allocation of decision rights and responsibilities, providing a reference point for examining their practical exercise.
**Enacted decision space**	Enacted decision space	What decisions can managers make and implement in practice?	Label retained to preserve the distinction between formally allocated authority and its practical exercise described in the programme findings.
**Bounded autonomy**	Bounds and contingencies	Under what organisational conditions does managers' scope for action vary?	Programme findings described limits arising from policies, finance, contracts and decisions at other levels. The broader formulation allows examination of how such conditions vary across circumstances and affect the exercise of authority.
**Support systems**	Support and resource systems	What support and resources do managers need to exercise delegated responsibilities?	Programme findings concerned administrative and professional expertise alongside operational resources. The broader label explicitly includes both support functions and the resources available for managerial action.
**Information infrastructure**	Information and feedback infrastructure	How do managers obtain information and feedback needed to assess situations and adjust decisions?	Programme findings concerned monitoring, information sharing and managerial feedback. Making feedback explicit extends comparison beyond information availability to how information supports review and adaptation.

Stage 1 analytical category	Provisional Stage 2 domain	Underlying organisational question	Basis for continuity or broadening
**Central coordination**	Coordination	How are interdependent activities connected across organisational levels and units?	Programme findings concerned shared priorities, logistics and collaboration across units. The broader label allows examination of how these functions are organised through both vertical and horizontal arrangements, including their enabling and constraining effects.
**Role ambiguity**	Accountability, decision ownership and role clarity	How are responsibilities, decision rights and accountability allocated and aligned?	Programme findings described overlapping responsibilities and uncertainty about decision ownership. The broader formulation addresses the underlying allocation and alignment of authority and responsibility, allowing comparison of both unclear and clearly specified arrangements.

Note. The domain formulations drew on the Stage 1 findings and their interpretation through Decision Space Theory and organisational theory. Broadening made the organisational questions underlying the original categories explicit and supported comparison across healthcare contexts. The domains provided a provisional organising structure for Stage 2 and remained open to revision and supplementation through the external review. Subsequent refinements arising from the external synthesis are distinct from this initial reformulation.
