# Supplementary material for: Dependent autonomy: developing and refining a theoretical construct of managerial autonomy in healthcare
Source: BMC Health Serv Res. 2026 Nov 9;26:1561. doi: 10.1186/s12913-026-16096-4 (PMC13749796; doi:10.1186/s12913-026-16096-4)
Supplement: Supplementary file 2 [file 12913_2026_16096_MOESM2_ESM.pdf]

Supplementary File 2A

Stage 2 review scope, searches and study selection

This supplement documents the external scoping review from its scope and search strategies to study selection. Supplementary File 2B presents the descriptive characteristics of all 78 included articles. Supplementary File 2C explains coding, comparison and construct refinement. The Stage 2 analysis workbook retains the complete selection register, evidence statements and source links.

Review scope

The preliminary construct from Stage 1 provided the starting point for the external review. The primary question was how organisational systems and contextual conditions shape the translation of formally delegated managerial authority into enacted decision space and managerial action in healthcare delivery organisations. The review mapped authority, its use or non-use, organisational conditions, contextual variation, proximal consequences and findings that challenged or extended the preliminary construct.

This condensed supplement summarises the recorded procedures. Conduct was informed by scoping-review methodology [27], with reporting guided by PRISMA-ScR [33] and PRISMA-S [34].

1. Inclusion and exclusion criteria

Table S2A.1 Detailed inclusion and exclusion criteria

Dimension	Inclusion criteria	Exclusion criteria and handling
**Population or unit of analysis**	Managers, management teams, boards or organisational units exercising managerial or organisational decision rights in healthcare delivery.	Patients or students alone; clinicians whose findings concern only clinical or professional autonomy; policy actors without a provider-level managerial link.
**Authority concept**	Explicit authority, autonomy, decision space, discretion, delegation, decentralisation, devolution or decision rights.	Leadership, engagement, culture or implementation studies without examination of authority, discretion, decision space or a clearly related managerial capacity to act.
**Analytical relevance**	An analysable relationship between authority and its enactment, organisational enablers or constraints, coordination, accountability, or proximal consequences for implementation, service organisation or patient flow.	Formal governance descriptions without findings on enactment or organisational conditioning; outcomes without an analysable link to authority or its organisational conditions.
**Healthcare context**	Healthcare delivery organisations or organised provider systems, including routine, reform and crisis settings.	Non-health sectors; macro-level policy studies without extractable provider- or manager-level findings; financing, insurance or regulation studies without relevant provider-level evidence.
**Empirical study design**	Primary qualitative, quantitative or mixed-methods studies, including case studies, longitudinal or process studies and empirical evaluations with sufficiently described methods and findings.	Secondary reviews, purely theoretical or conceptual papers, opinion pieces, commentaries, editorials and protocols.
**Publication type**	Full-length, peer-reviewed journal articles with sufficient methodological and results information for reliable charting.	Conference abstracts without a full report, book reviews, unpublished slide decks and publications with insufficient information for reliable charting.
**Language**	Full texts assessable in English.	Full texts that could not be assessed reliably in English.
**Date and geography**	Database inception to 31 August 2026; no geographical restriction.	No exclusion based solely on publication year or country within the search period.

Dimension	Inclusion criteria	Exclusion criteria and handling
**Separation from Stage 1**	Empirical evidence separate from the data underlying the four Stage 1 empirical studies. Other lead-author-linked reports were flagged and handled separately.	The four Stage 1 empirical studies and publications using the same empirical data. The preceding programme scoping review was not eligible as primary empirical evidence.
**Full-text availability**	Full text available for assessment through institutional access or other lawful retrieval routes.	Reports for which an accessible English-language full text was unavailable were recorded separately as not retrieved, rather than as substantive full-text exclusions.

Reviews and conceptual publications were retained separately for citation searching and conceptual positioning. HVHS/VBHC terms and patient-value outcomes were not mandatory eligibility criteria. Related external reports retained separate identifiers and were linked to avoid treating repeated findings as independent evidence.

Researcher roles

MO and AB independently conducted study selection, with UL adjudicating disagreements. AB and UL were independent of the empirical studies constituting the Stage 1 corpus. MO conducted data charting and initial analysis; AB checked charted information and reviewed coding, classifications and proposed refinements. UL independently reviewed disputed cases and adjudicated unresolved differences. Individual selection decisions and reasons were retained separately.

2. Search and deduplication

PubMed, CINAHL Complete (via EBSCOhost), and Web of Science Core Collection (Clarivate) were searched, with the final search conducted on 31 August 2026. Four concept blocks were combined using AND: healthcare delivery, managerial actors or units, authority or decision space, and organisational conditions. No publication-year restriction was specified. Database-specific search fields, filters, full queries and results are reported below. The final searches returned 494, 90 and 242 records, respectively, matching the 826 records entered into the review register.

Table S2A.2 Final database search results and deduplication

Database and platform	Fields in the review record	Records
**PubMed — NLM**	MeSH and title/abstract	494
**CINAHL Complete — EBSCOhost**	Title and abstract	90
**Web of Science Core Collection — Clarivate**	Topic	242
**Total database records**		826
**Duplicates removed**	DOI, PMID and normalised-title matching	216
**Unique records**		610

Backward and forward citation checking and identification of related reports informed retrieval and study-family mapping. These activities were not quantified as a separate identification stream. The counts describe the original final database search and include no subsequent search update.

3. Study selection

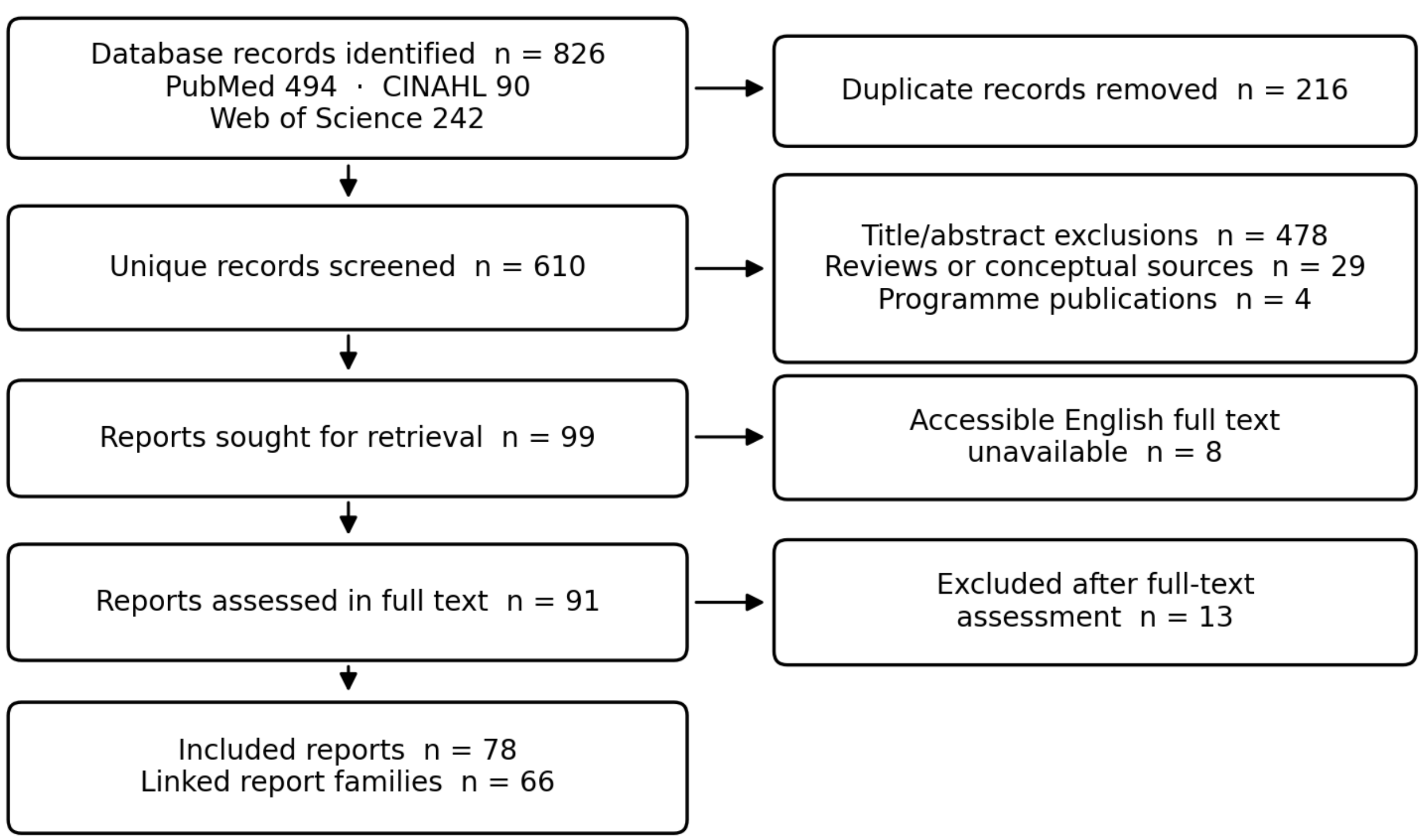

Figure S2A.1 Selection flow from the recorded search and retrieval sequence

Reviews and conceptual sources, and programme publications, were handled separately from the external empirical corpus. Eight reports lacked an accessible English full text; the record does not distinguish access from language restrictions for each report.

Selection decisions and full-text exclusions

The final corpus comprised 78 reports [8,35-111]. MO and AB agreed on inclusion and disagreements were adjudicated by UL. AB's review was completed, and the original individual decisions were retained.

Table S2A.3 Reports excluded after full-text assessment

Report and author	Title	Primary reason
**R024 Akol et al. 2015**	Health Managers' Views on the Status of National and Decentralized Health Systems for Child and Adolescent Mental Health in Uganda: A Qualitative Study	Authority, autonomy or decision space was not substantively examined.
**R052 Bosongo et al. 2024**	The Role of Provincial Health Administration in Supporting District Health Management Teams in the Democratic Republic of Congo: Eliciting an Initial Programme Theory of a Realist Evaluation	Initial programme-theory development without observed examination of provider-level enactment of managerial authority.
**R054 Cleary et al. 2013**	Resources, Attitudes and Culture: An Understanding of the Factors that Influence the Functioning of Accountability Mechanisms in Primary Health Care Settings	Secondary descriptive literature review with no new primary empirical data.
**R019 Davidson 1999**	British Columbia's Health Reform: "New Directions" and Accountability	Policy-historical/conceptual analysis without a reported empirical study design.
**R034 Holbeche 2011**	GP Consortia: Navigating Ambiguity to Produce Greater Public Value?	Not a primary empirical study of a healthcare delivery organisation.
**R046 Joseph et al. 2026**	Information Processing, Mutual Understanding, and Organization Design in Healthcare	Decentralisation was operationalised as redistribution of clinical ordering tasks rather than managerial or organisational decision rights.
**R037 Liu et al. 2016**	Development of the Chinese Version of the Hospital Autonomy Questionnaire: A Cross-Sectional Study in Guangdong Province	Instrument-development study without analysis of how formal authority is enacted or organisationally conditioned.
**R088 Malcolm & Barnett 1994**	New Zealand's health providers in an emerging market	Insufficiently described article-specific empirical methods and inseparable reliance on companion survey/interview material.
**R023 Mohammadi et al. 2019**	A Model Development for Hospital Holding Governance in the Iranian Social Security Organization	Governance model and measure development without analysis of enacted managerial authority.
**R071 Rechel, Duran & Saltman 2018**	What Is the Experience of Decentralized Hospital Governance in Europe? Ten Case Studies from Western Europe on Institutional and Accountability Arrangements	Rapid review and comparative country mapping without newly generated primary empirical data on enacted managerial authority.
**R018 Thuku et al. 2020**	Coordinating Health Workforce Management in a Devolved Context: Lessons from Kenya	No sufficiently described article-specific empirical analysis for reliable charting.
**R020 Vargas et al. 2016**	Barriers to Healthcare Coordination in Market-Based and Decentralized Public Health Systems: A Qualitative Study in Healthcare Networks of Colombia and Brazil	Coordination study without direct analysis of managerial/organisational authority or enacted decision space.
**R050 Winasti et al. 2023**	Entropy Optimization by Redesigning Organization in Hospital Operations	Alternative allocations of operational decision authority were modelled rather than observed in managerial practice.

One primary reason is shown for each of the 13 full-text exclusions. The workbook retains the original decision notes and secondary handling.

Retrieval limits

Reports without an accessible English full text

Report and author	Title
**R096 Ahmed 2024**	Analysing the Effectiveness of Decentralisation in Improving Health Services in Balochistan
**R098 Alexander & Fennell 1986**	PATTERNS OF DECISION-MAKING IN MULTIHOSPITAL SYSTEMS
**R095 Gilson, Kilima & Tanner 1994**	LOCAL-GOVERNMENT DECENTRALIZATION AND THE HEALTH SECTOR IN TANZANIA
**R093 Okamoto et al. 2009**	Developing institutional capacity of health service system management at the district level in rural Cambodia
**R092 Ortega-Díaz & Martín-Martín 2026**	Managerial autonomy and hospital performance: evidence from the Spanish National Health System
**R097 Puertas et al. 2018**	Integration of health service delivery networks in Honduras: a comparative assessment of theory and practice in five networks in the country
**R094 Spedo et al. 2009**	The challenge of decentralization of the Unified National Health System in large cities: the case of Sao Paulo, Brazil
**R099 Weil & Stam 1986**	TRANSITIONS IN THE HIERARCHY OF AUTHORITY IN HOSPITALS - IMPLICATIONS FOR THE ROLE OF THE CHIEF EXECUTIVE OFFICER

Search history and result counts

The following searches were run on 31 August 2026. Results for individual concept blocks and filtered variants overlap and must not be added. Only the final search from each database contributed to the total of 826 records.

Table S2A.4 Search sets and recorded result counts

Database	Set	Search or filter	Results	Time
PubMed	#1	Healthcare delivery; Abstract filter	5,182,020	12:22:01
PubMed	#2	Managerial actors or units; Abstract filter	1,853,057	12:22:29
PubMed	#3	Authority or decision space; Abstract filter	7,755	12:22:56
PubMed	#4	Organisational conditions; Abstract filter	257,858	12:23:41
PubMed	#5	#1 AND #2 AND #3 AND #4 AND english[lang]; Abstract filter	494	12:24:04
CINAHL	S1	Healthcare delivery	1,130,338	16:30:33
CINAHL	S2	Managerial actors or units	578,255	16:30:54
CINAHL	S3	Authority or decision space	1,447	16:31:09
CINAHL	S4	Organisational conditions	62,098	16:31:29
CINAHL	S5	S4 query with English Language filter	59,992	16:32:05
CINAHL	S6	(S1) AND (S2) AND (S3) AND (S4); English Language filter	90	16:33:34
Web of Science	#1	Healthcare delivery	3,118,092	19:04:33
Web of Science	#2	Managerial actors or units	4,302,310	19:05:18
Web of Science	#3	Authority or decision space	19,019	19:05:52
Web of Science	#4	Organisational conditions	816,125	19:06:19
Web of Science	#5	#1 AND #2 AND #3 AND #4	242	19:09:29

Search settings and source records

Times are reproduced as recorded: PubMed does not specify a time zone; CINAHL timestamps are UTC; Web of Science timestamps are UTC+02:00. PubMed used the Abstract filter for all five sets and english[lang] in the final query. CINAHL applied the English Language filter to S5 and S6. The Web of Science export does not display a separate language filter; English-language eligibility applied during study selection.

Source histories: PubMedSearchHistory (2).numbers; EBSCO-Search-history-metadata-08_31_2026 (1).pdf; search-history (1).pdf. All three histories are dated 31 August 2026. Final results match the review register: 494 + 90 + 242 = 826; after 216 duplicates were removed, 610 records remained for screening.

Full database search strategies

The queries below reproduce the user-entered search strings in the source histories. Line breaks and spacing have been standardised. Search-set numbers correspond to Table S2A.4; database filters and indexing coverage are specified for each database.

PubMed

NCBI PubMed; MeSH and title/abstract terms. The Abstract filter was active for sets #1–#5; the final query also specified English. No publication-year limit. Search time zone was not specified in the export.

#1

("Health Services Administration"[Mesh] OR "Delivery of Health Care"[Mesh] OR "Hospitals"[Mesh] OR "Primary Health Care"[Mesh] OR "Community Health Services"[Mesh] OR "Mental Health Services"[Mesh] OR healthcare[tiab] OR "health care"[tiab] OR "health service*"[tiab] OR "health system*"[tiab] OR hospital*[tiab] OR "primary care"[tiab] OR "primary health care"[tiab] OR "community health service*"[tiab] OR "community care"[tiab] OR "mental health service*"[tiab] OR "healthcare organisation*"[tiab] OR "healthcare organization*"[tiab] OR "health care organisation*"[tiab] OR "health care organization*"[tiab])

#2

("Health Facility Administrators"[Mesh] OR "Hospital Administrators"[Mesh] OR "Physician Executives"[Mesh] OR "Nurse Administrators"[Mesh] OR manager*[tiab] OR management[tiab] OR "clinical director*"[tiab] OR "medical director*"[tiab] OR "unit manager*"[tiab] OR "service manager*"[tiab] OR "middle manager*"[tiab] OR "senior manager*"[tiab] OR "management team*"[tiab] OR "hospital management"[tiab] OR "health service management"[tiab] OR "healthcare management"[tiab] OR "health care management"[tiab] OR "hospital administrator*"[tiab] OR "health administrator*"[tiab] OR "hospital executive*"[tiab] OR "healthcare executive*"[tiab] OR "health care executive*"[tiab] OR "hospital governance"[tiab] OR "healthcare governance"[tiab] OR "health care governance"[tiab])

#3

("dependent autonomy"[tiab] OR "decision space"[tiab] OR "managerial autonomy"[tiab] OR "management autonomy"[tiab] OR "organisational autonomy"[tiab] OR "organizational autonomy"[tiab] OR "hospital autonomy"[tiab] OR "facility autonomy"[tiab] OR "operational autonomy"[tiab] OR "earned autonomy"[tiab] OR "decision-making authority"[tiab] OR "decision making authority"[tiab] OR "decision authority"[tiab] OR "decision rights"[tiab] OR "delegated authority"[tiab] OR "formal authority"[tiab] OR "local autonomy"[tiab] OR "de jure autonomy"[tiab] OR "de facto autonomy"[tiab] OR "managerial discretion"[tiab] OR "management discretion"[tiab] OR ((decentralization[tiab] OR decentralisation[tiab] OR decentralized[tiab] OR decentralised[tiab] OR decentralizing[tiab] OR decentralising[tiab]) AND (management[tiab] OR managerial[tiab] OR authorit*[tiab] OR decision*[tiab])) OR ((delegation[tiab] OR delegated[tiab]) AND (authorit*[tiab] OR decision*[tiab] OR responsib*[tiab])) OR ((devolution[tiab] OR devolved[tiab]) AND (management[tiab] OR managerial[tiab] OR authorit*[tiab] OR decision*[tiab] OR responsib*[tiab])))

#4

("organisational capacity"[tiab] OR "organizational capacity"[tiab] OR "institutional capacity"[tiab] OR "managerial capacity"[tiab] OR "management capacity"[tiab] OR "administrative capacity"[tiab] OR "organisational support"[tiab] OR "organizational support"[tiab] OR "administrative support"[tiab] OR "management support"[tiab] OR "support system*"[tiab] OR "support function*"[tiab] OR "organisational system*"[tiab] OR "organizational system*"[tiab] OR "organisational infrastructure*"[tiab] OR "organizational infrastructure*"[tiab] OR "information infrastructure"[tiab] OR "information flow*"[tiab] OR "management information system*"[tiab] OR "performance information"[tiab] OR "feedback system*"[tiab] OR coordination[tiab] OR "coordination mechanism*"[tiab] OR "coordination arrangement*"[tiab] OR "coordination structure*"[tiab] OR "coordination system*"[tiab] OR "central coordination"[tiab] OR accountability[tiab] OR "accountability arrangement*"[tiab] OR "accountability structure*"[tiab] OR "accountability mechanism*"[tiab] OR "role clarity"[tiab] OR "role ambiguity"[tiab] OR "role conflict"[tiab] OR "governance arrangement*"[tiab] OR "enabling system*"[tiab] OR "implementation condition*"[tiab] OR "organisational enactment"[tiab] OR "organizational enactment"[tiab] OR "administrative burden"[tiab])

#5 Final combination

#1 AND #2 AND #3 AND #4 AND english[lang] Filter: Abstract. Results: 494.

The entered queries included “dependent autonomy”, “de jure autonomy” and “de facto autonomy” in #3, and “organisational enactment” and “organizational enactment” in #4. These phrases do not appear in the corresponding translated Search Details saved by PubMed; the source file retains those translations.

CINAHL Complete

CINAHL Complete via EBSCOhost; the export identifies the database as CINAHL. TI and AB denote title and abstract; N3 specifies proximity within three words in either order. Search mode: Find all my search terms. Expander: Apply equivalent subjects. No publication-year limit.

S1

(TI (healthcare OR "health care" OR "health service*" OR "health system*" OR hospital* OR "primary care" OR "primary health care" OR "community health service*" OR "community care" OR "mental health service*" OR "healthcare organisation*" OR "healthcare organization*" OR "health care organisation*" OR "health care organization*") OR AB (healthcare OR "health care" OR "health service*" OR "health system*" OR hospital* OR "primary care" OR "primary health care" OR "community health service*" OR "community care" OR "mental health service*" OR "healthcare organisation*" OR "healthcare organization*" OR "health care organisation*" OR "health care organization*"))

S2

(TI (manager* OR management OR "clinical director*" OR "medical director*" OR "unit manager*" OR "service manager*" OR "middle manager*" OR "senior manager*" OR "management team*" OR "hospital management" OR "health service management" OR "healthcare management" OR "health care management" OR "hospital administrator*" OR "health administrator*" OR "hospital executive*" OR "healthcare executive*" OR "health care executive*" OR "hospital governance" OR "healthcare governance" OR "health care governance") OR AB (manager* OR management OR "clinical director*" OR "medical director*" OR "unit manager*" OR "service manager*" OR "middle manager*" OR "senior manager*" OR "management team*" OR "hospital management" OR "health service management" OR "healthcare management" OR "health care management" OR "hospital administrator*" OR "health administrator*" OR "hospital executive*" OR "healthcare executive*" OR "health care executive*" OR "hospital governance" OR "healthcare governance" OR "health care governance"))

S3

(TI ("dependent autonomy" OR "decision space" OR "managerial autonomy" OR "management autonomy" OR "organisational autonomy" OR "organizational autonomy" OR "hospital autonomy" OR "facility autonomy" OR "operational autonomy" OR "earned autonomy" OR "decision-making authority" OR "decision making authority" OR "decision authority" OR "decision rights" OR "delegated authority" OR "formal authority" OR "local autonomy" OR "de jure autonomy" OR "de facto autonomy" OR "managerial discretion" OR "management discretion" OR (decentral* N3 manag*) OR (decentral* N3 authorit*) OR (decentral* N3 decision*) OR (delegat* N3 authorit*) OR (delegat* N3 decision*) OR (delegat* N3 responsib*) OR (devol* N3 manag*) OR (devol* N3 authorit*) OR (devol* N3 decision*) OR (devol* N3 responsib*)) OR AB ("dependent autonomy" OR "decision space" OR "managerial autonomy" OR "management autonomy" OR "organisational autonomy" OR "organizational autonomy" OR "hospital autonomy" OR "facility autonomy" OR "operational autonomy" OR "earned autonomy" OR "decision-making authority" OR "decision making authority" OR "decision authority" OR "decision rights" OR "delegated authority" OR "formal authority" OR "local autonomy" OR "de jure autonomy" OR "de facto autonomy" OR "managerial discretion" OR "management discretion" OR (decentral* N3 manag*) OR (decentral* N3 authorit*) OR (decentral* N3 decision*) OR (delegat* N3 authorit*) OR (delegat* N3 decision*) OR (delegat* N3 responsib*) OR (devol* N3 manag*) OR (devol* N3 authorit*) OR (devol* N3 decision*) OR (devol* N3 responsib*)))

S4

(TI ("organisational capacity" OR "organizational capacity" OR "institutional capacity" OR "managerial capacity" OR "management capacity" OR "administrative capacity" OR "organisational support" OR "organizational support" OR "administrative support" OR "management support" OR "support system*" OR "support function*" OR "organisational system*" OR "organizational system*" OR "organisational infrastructure*" OR "organizational infrastructure*" OR "information infrastructure" OR "information flow*" OR "management information system*" OR "performance information" OR "feedback system*" OR coordination OR accountability OR "role clarity" OR "role ambiguity" OR "role conflict" OR "governance arrangement*" OR "enabling system*" OR "implementation condition*" OR "organisational enactment" OR "organizational enactment" OR "administrative burden" OR (coordinat* N3 mechanism*) OR (coordinat* N3 arrangement*) OR (coordinat* N3 structur*) OR (coordinat* N3 system*) OR (accountab* N3 mechanism*) OR (accountab* N3 arrangement*) OR (accountab* N3 structur*) OR (accountab* N3 system*)) OR AB ("organisational capacity" OR "organizational capacity" OR "institutional capacity" OR "managerial capacity" OR "management capacity" OR "administrative capacity" OR "organisational support" OR "organizational support" OR "administrative support" OR "management support" OR "support system*" OR "support function*" OR "organisational system*" OR "organizational system*" OR "organisational infrastructure*" OR "organizational infrastructure*" OR "information infrastructure" OR "information flow*" OR "management information system*" OR "performance information" OR "feedback system*" OR coordination OR accountability OR "role clarity" OR "role ambiguity" OR "role conflict" OR "governance arrangement*" OR "enabling system*" OR "implementation condition*" OR "organisational enactment" OR "organizational enactment" OR "administrative burden" OR (coordinat* N3 mechanism*) OR (coordinat* N3 arrangement*) OR (coordinat* N3 structur*) OR (coordinat* N3 system*) OR (accountab* N3 mechanism*) OR (accountab* N3 arrangement*) OR (accountab* N3 structur*) OR (accountab* N3 system*)))

S5 English-language variant

The S4 query was repeated with the English Language filter, returning 59,992 results.

S6 Final combination

(S1) AND (S2) AND (S3) AND (S4) Filter: English Language = true. Results: 90.

Web of Science Core Collection

Web of Science Core Collection via Clarivate; TS denotes Topic and NEAR/3 specifies proximity within three words. Coverage recorded in the export: SCI and SSCI, 1900–2026; AHCI, 1975–2026; ESCI, 2005–2026. No publication-year restriction or separate language filter is shown in the exported searches.

#1

TS=(healthcare OR "health care" OR "health service*" OR "health system*" OR hospital* OR "primary care" OR "primary health care" OR "community health service*" OR "community care" OR "mental health service*" OR "healthcare organisation*" OR "healthcare organization*" OR "health care organisation*" OR "health care organization*")

#2

TS=(manager* OR management OR "clinical director*" OR "medical director*" OR "unit manager*" OR "service manager*" OR "middle manager*" OR "senior manager*" OR "management team*" OR "hospital management" OR "health service management" OR "healthcare management" OR "health care management" OR "hospital administrator*" OR "health administrator*" OR "hospital executive*" OR "healthcare executive*" OR "health care executive*" OR "hospital governance" OR "healthcare governance" OR "health care governance")

#3

TS=("dependent autonomy" OR "decision space" OR "managerial autonomy" OR "management autonomy" OR "organisational autonomy" OR "organizational autonomy" OR "hospital autonomy" OR "facility autonomy" OR "operational autonomy" OR "earned autonomy" OR "decision-making authority" OR "decision making authority" OR "decision authority" OR "decision rights" OR "delegated authority" OR "formal authority" OR "local autonomy" OR "de jure autonomy" OR "de facto autonomy" OR "managerial discretion" OR "management discretion" OR (decentral* NEAR/3 manag*) OR (decentral* NEAR/3 authorit*) OR (decentral* NEAR/3 decision*) OR (delegat* NEAR/3 authorit*) OR (delegat* NEAR/3 decision*) OR (delegat* NEAR/3 responsib*) OR (devol* NEAR/3 manag*) OR (devol* NEAR/3 authorit*) OR (devol* NEAR/3 decision*) OR (devol* NEAR/3 responsib*))

#4

TS=("organisational capacity" OR "organizational capacity" OR "institutional capacity" OR "managerial capacity" OR "management capacity" OR "administrative capacity" OR "organisational support" OR "organizational support" OR "administrative support" OR "management support" OR "support system*" OR "support function*" OR "organisational system*" OR "organizational system*" OR "organisational infrastructure*" OR "organizational infrastructure*" OR "information infrastructure" OR "information flow*" OR "management information system*" OR "performance information" OR "feedback system*" OR coordination OR accountability OR "role clarity" OR "role ambiguity" OR "role conflict" OR "governance arrangement*" OR "enabling system*" OR "implementation condition*" OR "organisational enactment" OR "organizational enactment" OR "administrative burden" OR (coordinat* NEAR/3 mechanism*) OR (coordinat* NEAR/3 arrangement*) OR (coordinat* NEAR/3 structur*) OR (coordinat* NEAR/3 system*) OR (accountab* NEAR/3 mechanism*) OR (accountab* NEAR/3 arrangement*) OR (accountab* NEAR/3 structur*) OR (accountab* NEAR/3 system*))

#5 Final combination

#1 AND #2 AND #3 AND #4 Results: 242.

Methodological references

[27] Peters MDJ, Marnie C, Tricco AC, Pollock D, Munn Z, Alexander L, McInerney P, Godfrey CM, Khalil H. Updated methodological guidance for the conduct of scoping reviews. JBI Evid Synth. 2020;18(10):2119-2126. doi:10.11124/JBIES-20-00167.

[33] Tricco AC, Lillie E, Zarin W, et al. PRISMA extension for scoping reviews (PRISMA-ScR): checklist and explanation. Ann Intern Med. 2018;169(7):467-473. doi:10.7326/M18-0850.

[34] Rethlefsen ML, Kirtley S, Waffenschmidt S, Ayala AP, Moher D, Page MJ, Koffel JB; PRISMA-S Group. PRISMA-S: an extension to the PRISMA Statement for Reporting Literature Searches in Systematic Reviews. Syst Rev. 2021;10:39. doi:10.1186/s13643-020-01542-z.
