# Supplementary material for: Dependent autonomy: developing and refining a theoretical construct of managerial autonomy in healthcare
Source: BMC Health Serv Res. 2026 Nov 9;26:1561. doi: 10.1186/s12913-026-16096-4 (PMC13749796; doi:10.1186/s12913-026-16096-4)
Supplement: Supplementary file 3 [file 12913_2026_16096_MOESM3_ESM.pdf]

Supplementary File 2B

Study level characteristics of the 78 included articles

The table describes the 78 articles included in the Stage 2 external scoping review of dependent autonomy [8,35–111], ordered by publication year (newest first) and author. Reference numbers correspond to the manuscript, and report IDs link each article to the review workbook.

Author (year) Reference / report ID	Country and setting	Journal	Title	Study design and data	System level	Main approach	Primary analytical focus
**Alawode et al. (2026)** [35] R013	Nigeria	Pan African Medical Journal	An Enabler or a Barrier: Implications of Local Government Autonomy for Effective Primary Health Care Reforms in Nigeria	Qualitative exploratory policy analysis with 288 key-informant interviews and desk review	Multilevel governance system	Local-government autonomy, fiscal capacity and primary-care reform governance	Decision space and decentralisation enactment
**Allawati et al. (2026)** [36] R012	Oman	Eastern Mediterranean Health Journal	An Evaluation of Three Decades of Health System Decentralisation in Oman	Document review and 35 key-informant interviews	National or multinational health system	Long-term evaluation of decentralised authority, governance and service performance	Decision space and decentralisation enactment
**Amollo et al. (2026)** [37] R025	Kenya	BMC Public Health	Financial Determinants of Effective Hypertension and Diabetes Care in Rural Primary Health Facilities in Kisumu, Kenya: A Mixed-Methods Study	Convergent parallel mixed-methods cross-sectional study	Facility or service-delivery unit	Facility financial autonomy, approval delays and chronic-care resource availability	Financial autonomy, budgeting and resource allocation
**Thongngun & Tiraphat (2026)** [106] R079	Thailand	BMC Health Services Research	Provincial Lessons on Long-Term Care Implementation in Thailand: Motivation and Managerial Capacities within a Decentralized Health System in a Middle-Income Country	Province-wide cross-sectional survey of 131 long-term-care system managers	Regional or subnational health system	Managerial processes, motivation and implementation performance in decentralised long-term care	Managerial capacity, support and adaptive implementation
**Dubé et al. (2025)** [51] R031	New Brunswick, Canada	INQUIRY: The Journal of Health Care Organization, Provision, and Financing	From Policy to Practice: A Qualitative Study on Reforms and Frontline Retention in Healthcare	Qualitative interviews with clinicians plus two managerial focus groups	Healthcare organisation, hospital or network	Frontline experience of reform, local autonomy and organisational accountability	Decision space and decentralisation enactment
**Gobezie et al. (2025)** [57] R086	Ethiopia	Scientific Reports	Healthcare governance practices and their determinants among public hospital managers in South Wollo zone, Northeast Ethiopia	Facility-based mixed-methods cross-sectional study with 182 public-hospital managers and 10 key informants	Healthcare organisation, hospital or network	Governance practice, managerial autonomy, feedback and political interference	Governance, accountability and organisational decision rights
**Jalilvand et al. (2025)** [63] R087	Iran	BMC Health Services Research	Hospital governance accountability challenges in Iran: a qualitative study	Qualitative study with 23 hospital administrators, presidents, directors and governance experts	Healthcare organisation, hospital or network	Hospital governance structures, accountability and constraints on managerial authority	Governance, accountability and organisational decision rights

Author (year) Reference / report ID	Country and setting	Journal	Title	Study design and data	System level	Main approach	Primary analytical focus
**Svensson (2025)** [105] R078	Sweden	BMC Health Services Research	Taking Sides or Bridging Worlds? Managerial Responses to Conflicts and Tensions between the Core Operations and the Administration in Healthcare	Qualitative study with 24 manager interviews across three regions	Healthcare organisation, hospital or network	Managerial boundary work between clinical operations and administration	Coordination, integration and network governance
**Binyaruka et al. (2024)** [43] R027	Tanzania	Health Systems & Reform	Does Provider Autonomy Work Well in Tanzania? Perspectives of Primary Care Facilities on Budget Execution under Direct Facility Financing and Factors Affecting Provider Autonomy in Singida Region	Qualitative interviews and group discussions in two councils	Facility or service-delivery unit	Provider financial autonomy and budget execution under direct facility financing	Financial autonomy, budgeting and resource allocation
**Birru et al. (2024)** [44] R028	Lesotho	BMC Health Services Research	Healthcare Workers' Views on Decentralized Primary Health Care Management in Lesotho: A Qualitative Study	Qualitative study with 21 semi-structured key-informant interviews	Multilevel governance system	Implementation of district-level management decentralisation across health-system functions	Decision space and decentralisation enactment
**Witter et al. (2024)** [111] R082	South Africa	PLOS ONE	Opening Decision Spaces: A Case Study on the Opportunities and Constraints in the Public Health Sector of Mpumalanga Province, South Africa	Participatory action research plus retrospective decision-space analysis of mixed programme data	Regional or subnational health system	Decision-space constraints and enablers across provincial health-system levels	Decision space and decentralisation enactment
**Ayim et al. (2023)** [39] R084	Ghana	Frontiers in Public Health	Why district assemblies disburse resources to district health systems for service delivery at district level in the context of decentralization: a comparative study of two districts in the Volta Region of Ghana	Mixed-methods comparative multiple-case study of two districts using documents, routine data and 24 key-informant interviews	Regional or subnational health system	District-assembly decision space and resource disbursement to health services	Financial autonomy, budgeting and resource allocation
**Björk et al. (2023)** [45] R051	Sweden	Frontiers in Psychology	Under Pressure – The Working Situation of Swedish Healthcare Managers during the First Wave of COVID-19	Mixed-methods survey and qualitative content analysis of open-ended responses	Healthcare organisation, hospital or network	Managerial authority, role clarity and organisational support during crisis	Managerial capacity, support and adaptive implementation
**Feldhaus et al. (2023)** [54] R032	India	Social Science & Medicine	Examining Decentralization and Managerial Decision Making for Child Immunization Program Performance in India	Structured manager interviews with factor analysis and structural equation modelling	Multilevel governance system	Decision space, capacity, accountability and immunisation programme performance	Decision space and decentralisation enactment
**Rodríguez et al. (2023)** [97] R073	Kenya, Malawi and Uganda	Health Policy and Planning	Political Economy Analysis of Subnational Health Management in Kenya, Malawi and Uganda	Cross-country political-economy analysis using documents and 112 key-informant interviews	Multilevel governance system	Political economy of subnational management environments across three countries	Decision space and decentralisation enactment
**Tsofa et al. (2023)** [109] R080	Kenya	PLOS Global Public Health	Political Economy Analysis of Sub-National Health Sector Planning and Budgeting: A Case Study of Three Counties in Kenya	Political-economy case study with 32 key-informant interviews	Regional or subnational health system	Political economy of county planning, budgeting and resource allocation	Financial autonomy, budgeting and resource allocation

Author (year) Reference / report ID	Country and setting	Journal	Title	Study design and data	System level	Main approach	Primary analytical focus
**Chama-Chiliba et al. (2022)** [48] R030	Zambia	Health Policy Open	Effects of Performance-Based Financing on Facility Autonomy and Accountability: Evidence from Zambia	Difference-in-differences and synthetic-control analysis using a three-arm cluster-randomised trial	Facility or service-delivery unit	Effects of performance-based financing on facility autonomy and accountability	Financial autonomy, budgeting and resource allocation
**Kesale, Mahonge & Muhanga (2022)** [65] R062	Tanzania	PLOS ONE	The Quest for Accountability of Health Facility Governing Committees Implementing Direct Health Facility Financing in Tanzania: A Supply-Side Experience	Cross-sectional mixed-methods study in 32 facilities	Facility or service-delivery unit	Governing-committee accountability under direct facility financing	Governance, accountability and organisational decision rights
**Kielmann et al. (2021)** [66] R021	South Africa	Health Policy and Planning	'We Had to Manage What We Had on Hand, in Whatever Way We Could': Adaptive Responses in Policy for Decentralized Drug-Resistant Tuberculosis Care in South Africa	Grounded-theory study using interviews and small-group discussions across three provinces	Multilevel governance system	Adaptive managerial responses to decentralised drug-resistant tuberculosis implementation	Managerial capacity, support and adaptive implementation
**Heerdegen et al. (2020)** [59] R045	Ghana	Frontiers in Public Health	How Does District Health Management Emerge Within a Complex Health System? Insights for Capacity Strengthening in Ghana	Qualitative study with 21 semi-structured interviews across six districts and regional/NGO levels	Regional or subnational health system	Systemic conditions shaping district managers' authority and capacity to act	Managerial capacity, support and adaptive implementation
**Liwanag & Wyss (2020)** [77] R067	Philippines	BMC Health Services Research	Who Should Decide for Local Health Services? A Mixed Methods Study of Preferences for Decision-Making in the Decentralized Philippine Health System	Mixed-methods study: 24 survey responses plus the same 27 interviews	Multilevel governance system	Preferred allocation and possible recentralisation of local decision authority	Decision space and decentralisation enactment
**Munywoki et al. (2020)** [88] R069	Kenya	International Journal for Equity in Health	Tracking Health Sector Priority Setting Processes and Outcomes for Human Resources for Health, Five Years after Political Devolution: A County-Level Case Study in Kenya	Mixed-methods county case study using policy documents, HR data and interviews	Regional or subnational health system	County decision space, capacity and accountability in human-resource priority setting	Human-resource authority and workforce management
**Duran et al. (2019)** [52] R011	Romania	International Journal of Health Policy and Management	Assessment of Public Hospital Governance in Romania: Lessons From 10 Case Studies	Ten-hospital governance assessment using interviews, questionnaires and documents	Healthcare organisation, hospital or network	Alignment of hospital responsibilities, managerial autonomy and governance capacity	Governance, accountability and organisational decision rights
**Kigume & Maluka (2019)** [68] R005	Tanzania	International Journal of Health Policy and Management	Decentralisation and Health Services Delivery in 4 Districts in Tanzania: How and Why Does the Use of Decision Space Vary Across Districts?	Qualitative comparative four-district decision-space study	Regional or subnational health system	Cross-district variation in enacted decision space and local innovation	Decision space and decentralisation enactment
**Kigume & Maluka (2019)** [69] R035	Tanzania	International Journal of Health Planning and Management	Health Sector Decentralisation in Tanzania: Analysis of Decision Space in Human Resources for Health Management	Qualitative multi-district interviews, focus groups and document analysis	Regional or subnational health system	Decision space for recruitment, deployment, promotion and incentives	Human-resource authority and workforce management

Author (year) Reference / report ID	Country and setting	Journal	Title	Study design and data	System level	Main approach	Primary analytical focus
**Kuwawenaruwa et al. (2019)** [71] R010	Tanzania	International Journal of Health Planning and Management	Bank Accounts for Public Primary Health Care Facilities: Reflections on Implementation from Three Districts in Tanzania	Qualitative three-district case study with focus groups and interviews	Facility or service-delivery unit	Facility bank accounts, local financial discretion and accountability	Financial autonomy, budgeting and resource allocation
**Liwanag & Wyss (2019)** [76] R066	Philippines	Health Research Policy and Systems	Optimising Decentralisation for the Health Sector by Exploring the Synergy of Decision Space, Capacity and Accountability: Insights from the Philippines	Qualitative reanalysis/framework synthesis of the same 27 interviews	Multilevel governance system	Synergy of decision space, capacity and accountability across health functions	Decision space and decentralisation enactment
**Sumah & Baatiema (2019)** [104] R003	Ghana	International Journal of Health Policy and Management	Decentralisation and Management of Human Resource for Health in the Health System of Ghana: A Decision Space Analysis	Mixed-methods cross-sectional survey and document analysis	Multilevel governance system	Decision space for devolved human-resource management across organisational levels	Human-resource authority and workforce management
**Do et al. (2018)** [50] R056	Vietnam	Journal of Public Health Management and Practice	Strengthening Public Health Management Capacity in Vietnam: Preparing Local Public Health Workers for New Roles in a Decentralized Health System	Descriptive programme evaluation/retrospective review of training and workplace projects	Multilevel governance system	Project-based management development for decentralised public-health responsibilities	Managerial capacity, support and adaptive implementation
**Kigume & Maluka (2018)** [67] R036	Tanzania	International Journal of Health Planning and Management	Health Sector Decentralisation in Tanzania: How Do Institutional Capacities Influence Use of Decision Space?	Qualitative four-district interviews, focus groups and document analysis	Regional or subnational health system	Institutional capacity as a condition for using delegated decision space	Managerial capacity, support and adaptive implementation
**Kigume, Maluka & Kamuzora (2018)** [70] R004	Tanzania	International Journal of Health Planning and Management	Decentralisation and health services delivery in Tanzania: Analysis of decision space in planning, allocation, and use of financial resources	Qualitative multi-district decision-space study using interviews, focus groups and documents	Regional or subnational health system	Decision space in planning, allocation and use of financial resources	Financial autonomy, budgeting and resource allocation
**Liwanag & Wyss (2018)** [75] R065	Philippines	PLOS ONE	What Conditions Enable Decentralization to Improve the Health System? Qualitative Analysis of Perspectives on Decision Space after 25 Years of Devolution in the Philippines	Qualitative framework-method analysis of 27 decision-maker interviews	Multilevel governance system	Conditions enabling effective decentralised decision-making across health functions	Decision space and decentralisation enactment
**McCollum et al. (2018)** [83] R068	Kenya	Health Policy and Planning	Priority Setting for Health in the Context of Devolution in Kenya: Implications for Health Equity and Community-Based Primary Care	Large qualitative multicounty study with interviews and focus groups	Regional or subnational health system	Devolution, political power and priority setting for equity and community health	Planning, priority setting and service organisation
**Nxumalo et al. (2018)** [91] R015	Kenya and South Africa	BMJ Global Health	Accountability Mechanisms and the Value of Relationships: Experiences of Front-Line Managers at Subnational Level in Kenya and South Africa	Comparative qualitative multisite synthesis from embedded health-system research	Regional or subnational health system	Relational and bureaucratic accountability in subnational management	Governance, accountability and organisational decision rights
**Saxena et al. (2018)** [98] R090	Canada	Leadership in Health Services	Structure of health-care dyad leadership: an organization's experience	Mixed-methods organisational study of 32 dyad leaders plus five senior-leader interviews	Healthcare organisation, hospital or network	Distribution of authority, responsibility and accountability in dyad leadership	Governance, accountability and organisational decision rights

Author (year) Reference / report ID	Country and setting	Journal	Title	Study design and data	System level	Main approach	Primary analytical focus
**Barasa et al. (2017)** [41] R026	Kenya	Social Science & Medicine	Hospitals as Complex Adaptive Systems: A Case Study of Factors Influencing Priority Setting Practices at the Hospital Level in Kenya	Two-hospital qualitative case study with 72 interviews, documents and observations	Healthcare organisation, hospital or network	Hospital priority setting under constrained autonomy, finance and leadership capacity	Planning, priority setting and service organisation
**Erchick et al. (2017)** [53] R057	Nigeria	International Journal of Health Policy and Management	Understanding Internal Accountability in Nigeria's Routine Immunization System: Perspectives From Government Officials at the National, State, and Local Levels	Semi-structured interview study across national, state, local and facility levels	Multilevel governance system	Internal accountability, role clarity and managerial problem-solving authority	Governance, accountability and organisational decision rights
**Henriksson et al. (2017)** [60] R033	Uganda	BMC Health Services Research	Enablers and Barriers to Evidence-Based Planning in the District Health System in Uganda: Perceptions of District Health Managers	Qualitative key-informant interview study in two districts	Regional or subnational health system	Decision space, political power and evidence-based district planning	Planning, priority setting and service organisation
**Islam (2017)** [61] R061	Bangladesh	Asia Pacific Journal of Health Management	The Influence of Politics on Accountability of Health Professionals in Bangladesh: An Analysis of the Quality of Health Service Delivery	Qualitative case study with 68 interviews and five focus groups	Multilevel governance system	Political authority, decentralisation and accountability in local health services	Governance, accountability and organisational decision rights
**Nyikuri et al. (2017)** [93] R022	Kenya	International Journal for Equity in Health	"We Are Toothless and Hanging, but Optimistic": Sub-County Managers' Experiences of Rapid Devolution in Coastal Kenya	Qualitative learning-site case study with observation and 15 in-depth interviews	Regional or subnational health system	Sub-county managerial autonomy, accountability and coping during rapid devolution	Decision space and decentralisation enactment
**Tsofa et al. (2017)** [107] R049	Kenya	International Journal for Equity in Health	Devolution and Its Effects on Health Workforce and Commodities Management: Early Implementation Experiences in Kilifi County, Kenya	Qualitative decision-space case study with documents, interviews and observations	Regional or subnational health system	County decision space and capacity for workforce and commodities management	Human-resource authority and workforce management
**Tsofa et al. (2017)** [108] R091	Kilifi County, Kenya	International Journal for Equity in Health	How does decentralisation affect health sector planning and financial management? a case study of early effects of devolution in Kilifi County, Kenya	Qualitative learning-site case study with 28 interviews, documents, observation and reflective practice	Regional or subnational health system	County planning, budgeting and financial management after rapid devolution	Financial autonomy, budgeting and resource allocation
**Geng, Yu & Chen (2016)** [55] R058	Laos	International Journal of Health Planning and Management	Preparing for the Introduction of Hospital Autonomy in Laos: An Assessment of Current Situation and Suggestions for Policy-Making	Hospital-autonomy assessment using key-informant interviews, focus groups and secondary data	National or multinational health system	Current hospital governance, informal autonomy and accountability readiness	Governance, accountability and organisational decision rights
**George et al. (2016)** [56] R059	Nigeria	Health Policy and Planning	Sparking, Supporting and Steering Change: Grounding an Accountability Framework with Viewpoints from Nigerian Routine Immunization and Primary Health Care Government Officials	Framework development plus open-ended questionnaires from 36 officials at three workshops	Multilevel governance system	Accountability framework grounded in authority, capability and managerial discretion	Governance, accountability and organisational decision rights

Author (year) Reference / report ID	Country and setting	Journal	Title	Study design and data	System level	Main approach	Primary analytical focus
**Kwamie et al. (2016)** [73] R064	Ghana	Health Policy and Planning	The Path Dependence of District Manager Decision-Space in Ghana	Theory-driven historical case study using literature, documents and key-informant interviews	National or multinational health system	Historical path dependence in expansion and contraction of district decision space	Decision space and decentralisation enactment
**Mohammed et al. (2016)** [87] R001	Fiji	International Journal of Health Policy and Management	Decentralisation of Health Services in Fiji: A Decision Space Analysis	Decision-space case study using policy/document analysis and empirical assessment	Regional or subnational health system	Functional assessment of administrative authority under health-service deconcentration	Decision space and decentralisation enactment
**Panda, Zodpey & Thakur (2016)** [95] R070	India	BMC Health Services Research	Local Self Governance in Health – A Study of Its Functioning in Odisha, India	Cross-sectional multi-district survey/interview study of 112 governing-body members	Facility or service-delivery unit	Local governing-body roles, involvement and determinants of decision-making	Governance, accountability and organisational decision rights
**Waweru et al. (2016)** [110] R081	Kenya	Health Policy and Planning	Tracking Implementation and (Un)Intended Consequences: A Process Evaluation of an Innovative Peripheral Health Facility Financing Mechanism in Kenya	Theory-of-change-based mixed-methods process evaluation	Facility or service-delivery unit	Facility financing, local decision-making and unintended accountability burdens	Financial autonomy, budgeting and resource allocation
**Bossert, Mitchell & Janjua (2015)** [46] R044	Pakistan	Health Systems & Reform	Improving Health System Performance in a Decentralized Health System: Capacity Building in Pakistan	Longitudinal comparative district surveys, 2006/2007 and 2009	Regional or subnational health system	Longitudinal effects of capacity building on decision space and performance	Decision space and decentralisation enactment
**Kwamie, Agyepong & van Dijk (2015)** [72] R063	Ghana	Health Systems & Reform	What Governs District Manager Decision Making? A Case Study of Complex Leadership in Dangme West District, Ghana	Theory-driven embedded case study using documents, participant observation and interviews	Regional or subnational health system	Hierarchical authority, power and trust in district managerial decision-making	Decision space and decentralisation enactment
**McDermott et al. (2015)** [84] R038	Ireland, the Netherlands and the United Kingdom	Human Resource Management	From Bipartite to Tripartite Devolved HRM in Professional Service Contexts: Evidence from Hospitals in Three Countries	Comparative qualitative study using secondary data and 128 interviews in nine hospitals	Healthcare organisation, hospital or network	Tripartite allocation and coordination of devolved human-resource responsibilities	Human-resource authority and workforce management
**Nyikuri et al. (2015)** [92] R006	Kenya	PLOS ONE	Crises and Resilience at the Frontline—Public Health Facility Managers under Devolution in a Sub-County on the Kenyan Coast	Qualitative learning-site case study with interviews and observations	Facility or service-delivery unit	Frontline facility-manager roles, accountabilities and resilience under devolution	Decision space and decentralisation enactment
**Nyland & Pettersen (2015)** [94] R048	Norway	International Journal of Public Sector Management	Hybrid Controls and Accountabilities in Public Sector Management: Three Case Studies in a Reforming Hospital Sector	Longitudinal multiple-case study using interviews, documents and survey data	Healthcare organisation, hospital or network	Hybrid controls, accountability and changing organisational decision space	Governance, accountability and organisational decision rights
**Baird et al. (2014)** [40] R007	United States	Health Care Management Review	Corporate Governance and the Adoption of Health Information Technology within Integrated Delivery Systems	Cross-sectional retrospective database study with multivariable regression	Healthcare organisation, hospital or network	Centralisation of information-technology decision rights and technology adoption	Information infrastructure and decision support

Author (year) Reference / report ID	Country and setting	Journal	Title	Study design and data	System level	Main approach	Primary analytical focus
**Büchner et al. (2014)** [47] R053	Germany	Health Care Management Review	The Impact of the Board's Strategy-Setting Role on Board–Management Relations and Hospital Performance	Cross-sectional survey paired across boards and hospital executives, with objective data and structural equation modelling	Healthcare organisation, hospital or network	Board strategy-setting, managerial autonomy and board-management collaboration	Governance, accountability and organisational decision rights
**Mkoka et al. (2014)** [86] R041	Tanzania	BMC Health Services Research	Governing the Implementation of Emergency Obstetric Care: Experiences of Rural District Health Managers, Tanzania	Qualitative organisational case study with interviews, focus group, documents and observation	Multilevel governance system	District governance, partnerships and constraints in policy implementation	Governance, accountability and organisational decision rights
**Sucaldito et al. (2014)** [103] R077	Philippines	International Journal of Public Health	The Philippines Field Management Training Program: Strengthening Management Capacity in a Decentralized Public Health System	Retrospective descriptive programme and applied-project review	National or multinational health system	Management training and workplace projects for new decentralised roles	Managerial capacity, support and adaptive implementation
**Kawonga, Fonn & Blaauw (2013)** [64] R014	South Africa	Global Health Action	Administrative Integration of Vertical HIV Monitoring and Evaluation into Health Systems: A Case Study from South Africa	Cross-sectional survey/interview study of 51 managers	Multilevel governance system	Exercised authority over vertical-programme information and data use	Information infrastructure and decision support
**Shayo, Mboera & Blystad (2013)** [100] R074	Tanzania	BMC Health Services Research	Stakeholders' Participation in Planning and Priority Setting in the Context of a Decentralised Health Care System: The Case of Prevention of Mother-to-Child Transmission of HIV Programme in Tanzania	Qualitative study with interviews and focus groups	Regional or subnational health system	Donor and central priorities in district programme planning and priority setting	Planning, priority setting and service organisation
**Bernardes et al. (2012)** [42] R085	Brazil	Revista Latino-Americana de Enfermagem	Framing the difficulties resulting from implementing a Participatory Management Model in a public hospital	Historical-organisational qualitative hospital case study with 39 nursing-team interviews	Healthcare organisation, hospital or network	Implementation gap between participatory structure and shared decision-making	Governance, accountability and organisational decision rights
**Newbrander et al. (2012)** [90] R016	Liberia	International Journal of Health Planning and Management	A Tool for Assessing Management Capacity at the Decentralized Level in a Fragile State	Development and field application of a management-capacity assessment tool in six counties	Regional or subnational health system	Assessment tool for decentralised management capacity in fragile settings	Managerial capacity, support and adaptive implementation
**Shaikh et al. (2012)** [99] R043	Pakistan	Journal of the Pakistan Medical Association	Experience of Devolution in District Health System of Pakistan: Perspectives Regarding Needed Reforms	Qualitative interview study with district and system actors	Regional or subnational health system	District experiences of devolved authority, resources and accountability	Decision space and decentralisation enactment
**Bossert & Mitchell (2011)** [8] R029	Pakistan	Social Science & Medicine	Health Sector Decentralization and Local Decision-Making: Decision Space, Institutional Capacities and Accountability in Pakistan	Cross-sectional quantitative survey of 91 decision-makers in 17 districts	Regional or subnational health system	Cross-functional decision space, capacity and accountability in districts	Decision space and decentralisation enactment

Author (year) Reference / report ID	Country and setting	Journal	Title	Study design and data	System level	Main approach	Primary analytical focus
**Grafton, Abernethy & Lillis (2011)** [58] R060	Victoria, Australia	Management Accounting Research	Organisational Design Choices in Response to Public Sector Reforms: A Case Study of Mandated Hospital Networks	Comparative three-network organisational case study	Healthcare organisation, hospital or network	Delegation of decision rights and control design in mandated hospital networks	Coordination, integration and network governance
**Maluka et al. (2011)** [81] R047	Tanzania	Implementation Science	Implementing Accountability for Reasonableness Framework at District Level in Tanzania: A Realist Evaluation	Realist process evaluation using interviews, observations and documents	Regional or subnational health system	Context-mechanism evaluation of accountability-based district priority setting	Planning, priority setting and service organisation
**Maluka et al. (2010)** [80] R040	Tanzania	Social Science & Medicine	Decentralized Health Care Priority-Setting in Tanzania: Evaluating against the Accountability for Reasonableness Framework	Qualitative baseline case study with interviews, documents and observation	Regional or subnational health system	Formal versus enacted participation and accountability in district priority setting	Planning, priority setting and service organisation
**Marchal, Dedzo & Kegels (2010)** [82] R017	Ghana	BMC Health Services Research	A Realist Evaluation of the Management of a Well-Performing Regional Hospital in Ghana	Realist mixed-methods organisational case study	Healthcare organisation, hospital or network	Human-resource management practices, support mechanisms and organisational commitment	Managerial capacity, support and adaptive implementation
**Qazi & Ali (2009)** [96] R072	Pakistan	Journal of the Pakistan Medical Association	Pakistan's Health Management Information System: Health Managers' Perspectives	Qualitative study with 30 in-depth/open-ended manager interviews	Regional or subnational health system	Health-information-system quality, feedback and conditions for managerial decisions	Information infrastructure and decision support
**Storey & Holti (2009)** [102] R076	England, United Kingdom	Journal of Health Organization and Management	Sense-Making by Clinical and Non-Clinical Executive Directors within New Governance Arrangements	Qualitative two-hospital case study with 17 executive interviews	Healthcare organisation, hospital or network	Executive sense-making of devolved service-line accountability and central control	Governance, accountability and organisational decision rights
**Lewis & Hinton (2008)** [74] R009	England, United Kingdom	Journal of Health Services Research & Policy	Citizen and Staff Involvement in Health Service Decision-Making: Have National Health Service Foundation Trusts in England Given Stakeholders a Louder Voice?	One-year organisational case study with interviews, observations and documents	Healthcare organisation, hospital or network	Governor roles, power-sharing and local accountability in foundation trusts	Governance, accountability and organisational decision rights
**Mutemwa (2006)** [89] R042	Zambia	Health Policy and Planning	HMIS and Decision-Making in Zambia: Re-Thinking Information Solutions for District Health Management in Decentralized Health Systems	Qualitative comparative case study tracing eight strategic decision processes	Regional or subnational health system	Use of formal and informal information in district strategic decisions	Information infrastructure and decision support
**Jack (2003)** [62] R008	Nicaragua	Health Policy and Planning	Contracting for Health Services: An Evaluation of Recent Reforms in Nicaragua	Policy/economic evaluation using reform documents and indicative empirical autonomy data	Multilevel governance system	Contracting, incentives and reallocation of authority in health-sector reform	Financial autonomy, budgeting and resource allocation
**McPake et al. (2003)** [85] R089	Bogotá, Colombia	Health Policy and Planning	Is the Colombian health system reform improving the performance of public hospitals in Bogotá?	Mixed-methods public-hospital reform evaluation using five-hospital time series, quality and satisfaction data, and 38 staff interviews	Healthcare organisation, hospital or network	Autonomous hospital reform, managerial discretion and organisational performance	Decision space and decentralisation enactment

Author (year) Reference / report ID	Country and setting	Journal	Title	Study design and data	System level	Main approach	Primary analytical focus
**Ssengooba et al. (2002)** [101] R075	Uganda	Public Administration and Development	What Could Be Achieved with Greater Public Hospital Autonomy? Comparison of Public and Private Not-for-Profit Hospitals in Uganda	Two-period mixed-methods comparative case study of paired hospitals	Multilevel governance system	Comparative hospital autonomy in staffing, procurement and fee setting	Decision space and decentralisation enactment
**Atkinson et al. (2000)** [38] R083	Northeast Brazil	Social Science & Medicine	Going down to the local: incorporating social organisation and political culture into assessments of decentralised health care	Comparative three-district ethnographic case study with prolonged fieldwork, interviews, observation, documents and service indicators	Regional or subnational health system	Local social organisation and political culture as mediators of decentralisation	Decision space and decentralisation enactment
**Conn, Jenkins & Touray (1996)** [49] R055	The Gambia	Health Policy and Planning	Strengthening Health Management: Experience of District Teams in The Gambia	Descriptive programme/process case of an 18-month management-strengthening project	Regional or subnational health system	Management strengthening under centrally constrained staffing, budgets and planning	Managerial capacity, support and adaptive implementation
**Malcolm & Barnett (1995)** [79] R039	New Zealand	Health Services Management Research	Decentralisation, Integration and Accountability: Perceptions of New Zealand's Top Health Service Managers	Representative postal survey of general and service managers	National or multinational health system	Manager perceptions of decentralised service management, integration and accountability	Coordination, integration and network governance
**Malcolm, Alp & Bryson (1994)** [78] R002	New Zealand	Health Services Management Research	Decentralisation of General Management within the New Zealand Health System	Document review and interviews with area health board general managers	National or multinational health system	Decentralisation of general management to service-level organisational units	Coordination, integration and network governance
