# Supplementary material for: Dependent autonomy: developing and refining a theoretical construct of managerial autonomy in healthcare
Source: BMC Health Serv Res. 2026 Nov 9;26:1561. doi: 10.1186/s12913-026-16096-4 (PMC13749796; doi:10.1186/s12913-026-16096-4)
Supplement: Supplementary file 4 [file 12913_2026_16096_MOESM4_ESM.pdf]

Supplementary File 2C

Stage 2 analysis and construct refinement

This supplement explains how findings from the external review were coded, compared and used to refine dependent autonomy. Supplementary File 2A documents the searches and selection; Supplementary File 2B lists all included articles and their characteristics. The Stage 2 analysis workbook contains the complete evidence matrix and linked records.

1 Deductive and inductive coding

The external review used hybrid deductive–inductive framework synthesis informed by best-fit framework synthesis [28]. The seven theory-informed categories used in Stage 1 were carried forward as provisional organising domains. Cross-study comparison in Stage 1 had generated three themes and a preliminary construct. Selected labels were broadened based on the Stage 1 findings and their interpretation through Decision Space Theory and organisational theory. This reformulation made the organisational questions underlying the categories more explicit and supported comparison of both enabling and constraining conditions across healthcare contexts. For example, "role ambiguity" became "accountability, decision ownership and role clarity" to encompass both unclear and clearly specified arrangements for allocating authority and responsibility. The mapping and rationale are presented in Supplementary File 1, Table S1.4.

An analytical evidence statement represented a distinct published empirical finding relevant to authority and its practical exercise. Eligible sources were results sections, tables, figures and supplements; discussion interpretations were included only when explicitly linked to reported findings. MO conducted initial coding.

Table S2C.1 Provisional organising domains and recorded statement counts

Code and domain	Analytical focus	Statements
D1 Formal decision authority	Decision rights, responsibilities or mandates allocated through law, policy, delegation, role or formal organisational structure.	32
D2 Enacted decision space	Authority or discretion actually used in practice, including use, non-use, adaptation, informal expansion or practical restriction.	77
D3 Bounds and contingencies	Limits, dependencies, sequencing conditions and contextual contingencies affecting whether authority can be used.	113
D4 Support and resource systems	Finance, staff, administrative support, competence, leadership, legitimacy, trust and other capacities supporting action.	87
D5 Information and feedback infrastructure	Data, information systems, analytics, feedback, reporting and learning loops relevant to managerial decisions.	21
D6 Coordination	Vertical, horizontal and inter-organisational arrangements integrating or aligning action across actors and levels.	67
D7 Accountability, decision ownership and role clarity	Answerability, enforcement, decision ownership, reporting lines, role definition and ambiguity.	85

Counts refer to primary domain assignments and sum to 482. Statements could also receive secondary domains and inductive codes. The workbook preserves original coding labels alongside the harmonised domain names.

The domains remained open to revision and supplementation during the external synthesis. Findings without a clear fit were retained and coded inductively. Inductive codes concerning legitimacy, working relationships and changing authority were examined across domains.

2 Comparison and classification

Findings were compared across reports, healthcare settings, managerial levels and study designs. Comparison addressed recurring relationships, contextual variation, contradictory findings, negative cases and rival explanations. The five classifications describe each evidence statement's relationship to the provisional construct. They apply to individual statements, and one publication may contribute statements in several classes. They are analytical categories used in this study, not a validated scoring instrument.

Table S2C.2 Relationship classifications and descriptive distribution

Classification	Meaning and use	Statements	Reports
Convergent	Supports a proposed component or relationship. Supports consideration of retention.	226	74
Qualifying	Specifies conditions, limits or contingencies. Clarifies scope and boundary conditions.	88	50
Divergent	Contradicts a proposed relationship or supports a rival explanation. Prompts reconsideration or reformulation.	10	9
Extending	Adds a condition, process or relationship. Informs expansion or further specification.	155	68
Indeterminate	Relevant but cannot be classified confidently. Retains uncertainty and identifies clarification needs.	3	3

The matrix contains 482 statements from 78 reports. Report counts indicate at least one statement in a class and overlap across classes. Neither statement nor report counts measure prevalence, conceptual importance, causal strength or independent reviewer agreement.

Context and overlap

Interpretation considered shared study families, organisational and managerial levels, healthcare settings, routine and crisis conditions, and the meaning of qualitative and quantitative findings. Apparent convergence was examined alongside counterexamples and contextual limits. The two corpora were analysed separately, and their counts were not pooled. Construct revisions did not use numerical vote-counting thresholds.

Study design and relevant limitations were charted descriptively. No formal critical appraisal, risk-of-bias assessment, quality scoring or quality-based weighting was undertaken. The synthesis mapped concepts, reported relationships and contextual variation for construct development [27,33].

Worked examples from evidence to interpretation

The examples below show different relationships to the provisional construct. They are shortened paraphrases of recorded evidence statements. IDs and PDF page numbers refer to the source versions recorded in the workbook. The contribution to refinement is the synthesis interpretation, separate from the reported finding.

Convergent example

Bossert & Mitchell (2011) [8]; R029; B2-R029-E01; PDF p. 1.

District officials reported different levels of exercised decision space despite operating under the same formal decentralisation rules.

Interpretation: Retain the distinction between formal authority and its practical exercise (FR01).

Qualifying example

Birru et al. (2024) [44]; R028; B2-R028-E03; PDF p. 11.

Formal district empowerment was not accompanied by a noticeable increase in district funding, limiting the practical resource base for decentralised action.

Interpretation: Specify accessible resources as a condition; formal empowerment alone does not provide them (FR04).

Divergent example

Mutemwa (2006) [89]; R042; B2-R042-E02; PDF p. 1.

Observed strategic decisions showed a weaker relationship between formal HMIS information and actual managerial decisions than normative models assumed.

Interpretation: Distinguish the presence of information infrastructure from information used in decisions (FR05).

Extending example

McDermott et al. (2015) [84]; R038; B2-R038-E03; PDF p. 1.

Senior professionals provided expertise, credibility and advocacy that made line managers' people-management decisions more legitimate in professional settings.

Interpretation: Consider recognised professional legitimacy as a condition cutting across domains (FR08).

Indeterminate example

Feldhaus et al. (2023) [54]; R032; B2-R032-E04; PDF p. 6.

Measured institutional capacity and accountability were not significantly associated with IMI effectiveness or efficiency.

Interpretation: Retain uncertainty. This finding does not by itself establish that capacity or accountability is irrelevant.

3 Construct refinement and consensus

Table S2C.3. Construct refinements and illustrative external findings.

The examples are paraphrases of published findings. Complete evidence links and review records are retained in the Stage 2 analysis workbook.

Component	Illustrative external findings	Refinement
**FR01**<br>**Formal decision authority**	Authority differed across functions and managerial levels in Oman (Allawati et al., 2026 [36]). District officials in Pakistan reported different levels of exercised decision space under the same formal decentralisation rules (Bossert & Mitchell, 2011 [8]).	**Retain and qualify.** Specify authority by function, level and actor. Distinguish its formal allocation from its practical exercise.
**FR02**<br>**Enacted decision space**	Hospital networks complied formally with collaboration requirements while retaining hospital-centred operating systems (Grafton et al., 2011 [58]). Central approval requirements prompted parallel information systems (Allawati et al., 2026 [36]). Semi-autonomous units also generated boundary conflicts and risks of prioritising their own services (Malcolm et al., 1994 [78]).	**Retain and broaden.** Include use, non-use, adaptation, deferral, informal expansion and override. Distinguish formal compliance from changes in practice.
**FR03**<br>**Bounds and contingencies**	The practical effects of decentralisation varied with local political relations, staff involvement and accountability arrangements (Atkinson et al., 2000 [38]). Greater measured decision space was associated with less improvement in an immunisation coverage gap (Feldhaus et al., 2023 [54]).	**Retain and extend.** Specify organisational, political, professional and temporal conditions affecting the scope and consequences of authority. Wider decision space does not necessarily imply better performance.
**FR04**<br>**Deployable organisational capacity**	District empowerment was not accompanied by a noticeable increase in funding (Birru et al., 2024 [44]). Devolved HR responsibilities depended on accessible specialist support (McDermott et al., 2015 [84]). Trust, communication and local initiative also supported implementation despite material shortages (Kielmann et al., 2021 [66]).	**Retain and specify availability.** Distinguish the presence of resources from their accessibility and usability. Include expertise and relational support alongside material resources.
**FR05**<br>**Information and feedback infrastructure**	Regional teams used local data analysis to seek staff and target supervision (Conn et al., 1996 [49]). Central controls prompted parallel data systems (Allawati et al., 2026 [36]). Formal health-information systems had a weaker relationship with actual decisions than normative models assumed (Mutemwa, 2006 [89]).	**Retain and extend.** Specify how information is interpreted and used in decisions, feedback and learning. The presence of a formal system does not establish its practical use.
**FR06**<br>**Coordination**	District–facility requisition routines improved the match between requested and supplied commodities (Birru et al., 2024 [44]). Devolved HR management involved relationships among HR practitioners, line managers and senior professionals (McDermott et al., 2015 [84]). Semi-autonomous units also experienced cross-boundary tensions (Malcolm et al., 1994 [78]).	**Retain and broaden.** Include formal arrangements and working relationships connecting interdependent actors. Consider integration alongside delay, fragmentation and competing priorities.

Component	Illustrative external findings	Refinement
**FR07 Accountability, decision ownership and role clarity**	Clearer authority levels and supportive supervision strengthened district management (Birru et al., 2024 [44]). Unclear roles impaired devolved HR management (McDermott et al., 2015 [84]). No overall relationship was found between decision space and accountability to elected officials (Bossert & Mitchell, 2011 [8]).	**Retain and specify duality.** Clarify the alignment of accountability, responsibilities and decision rights, including escalation arrangements. Greater decision space does not automatically imply stronger accountability.
**FR08 Recognised legitimacy**	Senior professionals provided credibility and advocacy supporting line managers' decisions (McDermott et al., 2015 [84]). Acceptance of a coordination mandate was linked to perceived efficiency and legitimacy gains (Grafton et al., 2011 [58]). Supportive relationships generated organisational commitment despite narrow formal authority (Marchal et al., 2010 [82]).	**Specify a cross-cutting condition.** Make explicit how professional, organisational and political recognition may affect the acceptance and use of authority across domains.
**FR09 Feedback and reconfiguration**	Action planning and local data use increased regional teams' involvement in planning, resource requests and coordination (Conn et al., 1996 [49]). Most respondents in a Philippine study preferred some form of recentralisation over retaining existing arrangements unchanged (Liwanag & Wyss, 2020 [77]).	**Specify a proposition for further examination.** Feedback from managerial experience and action may inform changes in decision rights and organisational arrangements. These examples do not establish that feedback produced such changes over time.

Note. "Retain", "qualify", "broaden", "extend" and "specify" describe synthesis decisions. Each refinement may draw on findings from several analytical classifications. FR01–FR07 concern the seven organising domains; FR08 specifies a cross-cutting condition; and FR09 states a proposition requiring further empirical examination. The examples illustrate the basis for conceptual refinement and do not establish causal effects.

Construct and propositions

Dependent autonomy is proposed as a theoretical construct describing the organisationally conditioned actionability of formally delegated managerial authority. The framework identifies possible underlying processes, including interpretation of mandates, resource mobilisation, information use and cooperation. Their causal operation was not established by this synthesis.

Proposition	Focus
P1	Formal authority is function-specific and distributed. Its allocation does not determine whether or how it can be exercised.
P2	Actionability depends on usable resources, support and information, coordination, recognised legitimacy and accountability aligned with responsibilities and decision rights.
P3	The consequences of actionable authority depend on its exercise and context. Greater scope for action does not necessarily improve organisational performance.
P4	Feedback from action, non-action and adaptations may expand, restrict or redistribute decision rights and organisational arrangements over time.

HVHS/VBHC provides an application context. Patient value remains a distal conceptual orientation; the review does not establish causal effects on patient outcomes, efficiency or experience.

Review and documentation

AB completed the independent review of coding, relationship classifications and proposed refinements. UL independently reviewed disputed cases and adjudicated unresolved differences. For study selection, MO and AB agreed on inclusion decisions, with disagreements adjudicated by UL.

References cited in this supplement

[8] Bossert TJ, Mitchell AD. Health sector decentralization and local decision-making: Decision space, institutional capacities and accountability in Pakistan. Social science & medicine (1982). 2011;72(1):39–48. doi: 10.1016/j.socscimed.2010.10.019

[27] Peters MDJ, Marnie C, Tricco AC, Pollock D, Munn Z, Alexander L, McInerney P, Godfrey CM, Khalil H. Updated methodological guidance for the conduct of scoping reviews. JBI Evid Synth. 2020;18(10):2119-2126. doi:10.11124/JBIES-20-00167.

[28] Carroll C, Booth A, Leaviss J, Rick J. Best fit framework synthesis: refining the method. BMC Med Res Methodol. 2013;13:37. doi:10.1186/1471-2288-13-37.

[33] Tricco AC, Lillie E, Zarin W, et al. PRISMA extension for scoping reviews (PRISMA-ScR): checklist and explanation. Ann Intern Med. 2018;169(7):467-473. doi:10.7326/M18-0850.

[36] Allawati NR, Mohamed N, Al Nadabi W. An evaluation of three decades of health system decentralisation in Oman. East Mediterr Health J. 2026;32(1):12-18. doi:10.26719/2026.32.1.12.

[38] Atkinson S, Medeiros RL, Oliveira PH, de Almeida RD. Going down to the local: incorporating social organisation and political culture into assessments of decentralised health care. Soc Sci Med. 2000;51(4):619-36. doi:10.1016/s0277-9536(00)00005-8.

[44] Birru E, Ndayizigiye M, Wanje G, Marole T, Smith PD, Koto M, et al. Healthcare workers' views on decentralized primary health care management in Lesotho: a qualitative study. BMC Health Serv Res. 2024;24(1):801. doi:10.1186/s12913-024-11279-3.

[49] Conn CP, Jenkins P, Touray SO. Strengthening health management: experience of district teams in The Gambia. Health Policy Plan. 1996;11(1):64-71. doi:10.1093/heapol/11.1.64.

[54] Feldhaus I, Chatterjee S, Clarke-Deelder E, Brenzel L, Resch S, Bossert TJ. Examining decentralization and managerial decision making for child immunization program performance in India. Soc Sci Med. 2023;317:115457. doi:10.1016/j.socscimed.2022.115457.

[58] Grafton J, Abernethy MA, Lillis AM. Organisational design choices in response to public sector reforms: a case study of mandated hospital networks. Manag Account Res. 2011;22(4):242-68. doi:10.1016/j.mar.2011.06.001.

[66] Kielmann K, Dickson-Hall L, Jassat W, Le Roux S, Moshabela M, Cox H, et al. 'We had to manage what we had on hand, in whatever way we could': adaptive responses in policy for decentralized drug-resistant tuberculosis care in South Africa. Health Policy Plan. 2021;36(3):249-59. doi:10.1093/heapol/czaa147.

[77] Liwanag HJ, Wyss K. Who should decide for local health services? A mixed methods study of preferences for decision-making in the decentralized Philippine health system. BMC Health Serv Res. 2020;20(1):305. doi:10.1186/s12913-020-05174-w.

[78] Malcolm L, Alp B, Bryson J. Decentralisation of general management within the New Zealand health system. Health Serv Manage Res. 1994;7(4):220-8. doi:10.1177/095148489400700402.

[82] Marchal B, Dedzo M, Kegels G. A realist evaluation of the management of a well-performing regional hospital in Ghana. BMC Health Serv Res. 2010;10:24. doi:10.1186/1472-6963-10-24.

[84] McDermott AM, Fitzgerald L, Van Gestel NM, Keating MA. From bipartite to tripartite devolved HRM in professional service contexts: evidence from hospitals in three countries. Hum Resour Manage. 2015;54(5):813-31. doi:10.1002/hrm.21728.

[89] Mutemwa RI. HMIS and decision-making in Zambia: re-thinking information solutions for district health management in decentralized health systems. Health Policy Plan. 2006;21(1):40-52. doi:10.1093/heapol/czj003.
